# A Space‐Dependent ‘Enzyme‐Substrate’ Type Probe based on ‘Carboxylesterase‐Amide Group’ for Ultrafast Fluorescent Imaging Orthotopic Hepatocellular Carcinoma

**DOI:** 10.1002/advs.202206681

**Published:** 2023-01-18

**Authors:** Ying Wen, Ning Jing, Min Zhang, Fangjun Huo, Zhuoyu Li, Caixia Yin

**Affiliations:** ^1^ Key Laboratory of Chemical Biology and Molecular Engineering of Ministry of Education Key Laboratory of Materials for Energy Conversion and Storage of Shanxi Province Institute of Molecular Science Shanxi University Taiyuan 030006 China; ^2^ State Key Laboratory of Component‐based Chinese Medicine Tianjin University of Traditional Chinese Medicine Tianjin 301617 China; ^3^ Research Institute of Applied Chemistry Shanxi University Taiyuan 030006 China; ^4^ Institute of Biotechnology Key Laboratory of Chemical Biology and Molecular Engineering of National Ministry of Education Shanxi University Taiyuan 030006 China

**Keywords:** carboxylesterase, enzyme probe, fluorescent response, orthotopic hepatocellular carcinoma

## Abstract

Fast and selective fluorescence imaging for a biomarker to related‐disease diagnosis remains a significant challenge due to complex physical environment. Human carboxylesterase (CE) is expected to be a potential biomarker of hepatocellular carcinoma (HCC) to improve the accuracy of diagnosis. However, existing probes for CE has slow response rate and low selectivity. Herein, the amide group is selected as CE‐responsive sites based on the “substrate‐hydrolysis enzymatic reaction” approach. From a series of off–on probes with leave groups in the amide unit, probe **J_Fast_** is screened with the optimal combination of rapid response rate and high selectivity toward CE. **J_Fast_** requires only 150 s to reach the maximum fluorescence at 676 nm in the presence of CE and free from the interference of other esterase. Computational docking simulations indicate the shortest distance between the CE and active site of **J_Fast_**. Cell and in vivo imaging present that the probe can turn on the liver cancer cells and tumor region precisely. Importantly, **J_Fast_** is allowed to specifically image orthotopic liver tumor rather than metastatic tumor and distinguish human primary liver cancer tissue from adjacent ones. This study provides a new tool for CE detection and promotes advancements in accurate HCC diagnosis.

## Graphical abstract

Based on the “substrate‐hydrolysis enzymatic reaction” approach, a fluorescent probe toward carboxylesterase with ultra‐rapid response rate is designed and developed, which is allowed to specifically image orthotopic liver tumor rather than metastatic tumor and distinguish human primary liver cancer tissue from adjacent ones.

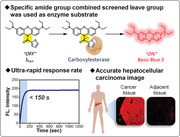

## 1 Introduction

Hepatocellular carcinoma (HCC) accounts for >80% of primary liver cancers worldwide.^[^ ^1^ ^]^ HCC exacts a heavy disease burden and has inferior survival rates (<5% during 5 years) because most HCC patients are diagnosed at later stages, restricting therapeutic options.^[^ ^1^, ^2^ ^]^ Accurate detection and differential diagnosis of early HCC is undoubtedly beneficial to improve patient survival. Compared to traditionally radiological diagnosis, involving multiphasic contrast computed tomography or magnetic resonance imaging, the detection of biomarkers associated with HCC in body fluids or tissues, is the most promising approach to improve diagnostic accuracy and to overcome the disadvantages of radiologically diagnostic strategies, such as frequent monitoring and radiation exposure.^[^ ^3^ ^]^ Although alpha‐fetoprotein (AFP) is currently the major serologic biomarker for HCC, it cannot efficiently distinguish HCC from other forms of liver disease in early diagnosis due to its low sensitivity and specificity.^[^ ^4^ ^]^ It has been shown that 80% of small HCC nodules do not display increased AFP levels.^[^ ^5^, ^6^ ^]^ Consequently, several other biomarkers have been suggested to complement AFP to increase the accuracy of HCC detection, such as des‐*γ*‐carboxyprothrombin, lectin‐bound AFP, osteopontin, Golgi protein‐73, etc. However, reports on the levels of these markers are sometimes conflicting.^[^ ^7^ ^]^ Therefore, it is necessary to validate additional candidates and establish optimal detection methods for potential biomarkers.

Human carboxylesterase (CE), one of the most abundant serine hydrolases distributed in the human liver, plays a role in the metabolism of xenobiotic compounds in the liver and is expected to be a novel biomarker candidate for HCC.^[^ ^3^, ^8^, ^9^ ^]^ A database searching research suggested that CE could serve as an excellent serologic indicator for hepatocyte injury.^[^ ^10^ ^]^ A recent study indicated that the levels of CE were significantly higher in patients with HCC than healthy donors and the other liver diseases based on ELISA tests in 208 patients with 57 HCC patients.^[^ ^8^ ^]^ The indiscriminating between HCC versus liver cirrhosis, the significance of CE differences was more remarkable than those of AFP.^[^ ^8^ ^]^

Commercial kits use acetate‐1‐naphthalene ester to perform a colorimetric assay to determine CE activity in in vitro analysis. As we know, fluorescence imaging based on fluorescent probes is allowed to perform in vitro, ex vivo, and in vivo images and provide an analysis with higher sensitivity.^[^ ^11^, ^12^, ^13^, ^14^, ^15^, ^16^, ^17^, ^18^, ^19^ ^]^ It has surfaced as a powerful tool for visualizing target analytes in complicated biological specimens owing to its real‐time detection, non‐destructive analysis and, especially, excellent sensitivity.

**Figure 1:**
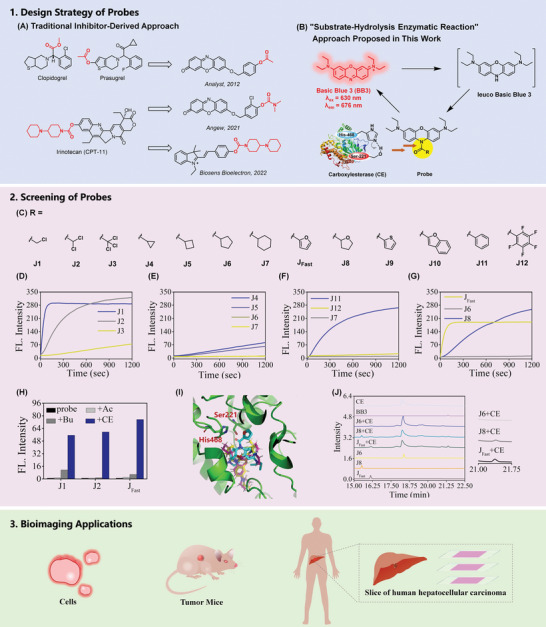
Probes for CE based on A) traditional inhibitor‐derived approach and B) “substrate‐hydrolysis enzymatic reaction” approach proposed in this paper. C) Designed probes for CE. D–G) Time‐dependent change of fluorescence emission intensity (*λ*_ex_ = 630 nm, *λ*_ex_ = 676 nm) of probes (5 µm) upon addition with CE (10 U mL^−1^). H) Selectivity tests of **J1**, **J2** and **J_Fast_** to CE. Fluorescence intensity 676 nm of 5 µm probes after addition of CE, AChE and BChE, upon excitation with 630 nm, respectively. I) Docked structures highlighting the interaction of probes in the active site of CE (PDB ID: 5A7H). **J_Fast_**, **J6** and **J8** structures are shown in cyan, magenta, and yellow, respectively. J) HPLC profiles of CE‐catalyzed hydrolysis of **J_Fast_**, **J6** and **J8**. Reactions are carried out for 5 min at 25 °C in PBS and monitored by analytical HPLC.

The design of fluorescent probes for CE to date has been based on “inhibitor‐derived” approach (**Figure** **1**A), a classic design method of enzyme‐probe. In this strategy, an active part or whole structure of classic inhibitor of enzyme were introduced and conjugated to fluorescent chromophore to regulate the release of fluorescence.^[^ ^20^, ^21^, ^22^, ^23^, ^24^ ^]^ CE tends to hydrolyze compounds containing the carboxylic ester bonds, such as clopidogrel, prasugrel, etc.^[^ ^25^ ^]^ From this, as early as in 2012, Ma's group developed a resorufin‐based fluorescence off–on probe through introduction of carboxylic ester bond as responsive sites to CE,^[^ ^26^ ^]^ as shown in Figure 1A. Upon reaction with CE, the carboxylic ester bond is cleaved by CE, leading to the release of resorufin and an increase of fluorescence intensity correspondingly. Since then, a series of probes that employed the carboxylic ester bonds as responsive sites have been reported and was used to investigate CE activity at physiological levels in ex vivo and in vivo (Table S1, Supporting Information).^[^ ^26^, ^27^, ^28^, ^29^, ^30^, ^31^, ^32^, ^33^ ^]^ Despite their wide use, the carboxylic ester recognition moieties easily suffer from the potential mutual interference of other esterases, such as acetylcholinesterase (AChE) and butyrylcholinesterase (BChE),^[^ ^34^, ^35^, ^36^ ^]^ which may seriously compromise the accurate measurement of CE activity in vivo. Recently, a probe with improved selectivity was designed and developed by Yoon's group based on carbamate unit as response group (Figure 1A), because author noticed irinotecan (CPT‐11) as a precursor of an anticancer drug containing a carbamate unit could be a substrate catalyzed by CE.^[^ ^37^ ^]^ The probe can avoid potential interference from AChE and BChE in monitoring CE. However, the probe requires circa 5 h to achieve maximum fluorescence emission in the presence of CE. Similarly, by incorporating bipiperidinyl into the merocyanine fluorophore, Ma's group reported another new fluorescent probe based on CPT‐11 inhibitor. Although the probe also presented high selectivity for CE and could avoid the interference of other esterase,^[^ ^38^ ^]^ the response rate performs imperfect. After addition of CE, maximum fluorescence emission was achieved for a long 5 h. These key studies provide strategies guidance and serve as practical cases in CE detection methodology, and indicated that a probe that combinate high selectivity and fast response rate for CE remain to be developed.

We noticed that CE are capability of hydrolyze chemicals containing a functional group such as amide.^[^ ^39^ ^]^ The critical “substrate‐hydrolysis enzymatic reaction” characteristics of CE inspire us to employ amide group as a specific recognition unit for CE (Figure 1B). We selected basic blue 3 (**BB3**) as fluorophores based on their excellent fluorescence properties known from our previous live‐cell/tissue imaging and clinical blood analysis.^[^ ^40^ ^]^ And, a series of off–on probes having different R substituents in the amide group (Figure 1C) were designed and synthesized to screen the probe with superior analytical performance and provide a systematic and comprehensive view on whether and how different components (R group, Figure 1B) design might affect the probe's response to CE. The probe **J_Fast_** (Figure 1C) exhibited the optimal property combination of selectivity and response rate toward CE. **J_Fast_** only requires 150 s to reach the maximum fluorescence in presence of CE and effectively eliminate the interference of AChE and BChE. Moreover, **J_Fast_** was cell membrane permeable and was successfully applied to monitor the real activities of CE in liver cancer cells. Importantly, selective imaging of CE activity in orthotopic liver tumor mice, rather than in metastatic tumor was performed. **J_Fast_** also was allowed to distinguish human primary liver cancer tissue from adjacent ones with a marked fluorescence enhancement. These results revealed the potential clinical application value of **J_Fast_**, and promoted the study of physiological and pathological processes related to HCC.

## 2 Results and Discussion

### 2.1 Design and Screening of Probes

A fluorescent probe for an enzyme should consist of two elements: fluorescence reporting and detecting parts. As reported in our previous work, the reduced form of **BB3** is non‐fluorescent. At the same time, oxidation of **BB3** could emit intensive fluorescence in the near‐infrared region majored at 676 nm, upon excitation with 630 nm. Thus, leuco basic blue 3 was allowed as scaffolds containing an amide group to construct near‐infrared “turn‐on” probes for the detection of CE.

A series of off–on probes having different R substituents in the amide group (Figure 1C) were designed and synthesized to screen the probe with superior analytical performance. The synthetic details (Scheme S1, Supporting Information) and procedures for probes are described in the Supporting Information, and the chemical structures of the molecules are fully characterized by HR‐MS, ^1^H NMR, and ^13^C NMR, respectively, as shown in Figures S1–S38 (Supporting Information).

The probes **J1**‐**J3** (Figure 1C) composed of a different number of chlorine‐substituents trigger groups (from one to three‐chlorine substituents) are constructed by the incorporation of a chloracetyl, dichloracetyl or thrichloroacetyl into leuco **BB3** via the amide bond, respectively. Fluorescent dynamic change curves are shown in Figure 1D–G. The fluorescence intensity at 676 nm (*F*_676_) of **J1** rapidly and drastically increased, upon exaction at 630 nm (Figure 1D). In sharp contrast to **J1**, the addition of CE did induce slight increase in *F*_676_ of **J3** within 1200 s. The fluorescent dynamics curve of **J2** is between **J1** and **J3**. Under the same conditions, *F*_676_ of **J2** slowly increases, and enters the platform region after 1200 s. In the selectivity tests (Figure 1H), **J2** produced better response toward CE (60‐fold increase) but had almost no response to AChE and BChE (<1.5‐fold increase). While, under the same conditions, the addition of BChE also induce an 11‐fold rise of **J1** in *F*_676_, compared to 55‐fold increase owing to the addition of CE, which indicated that **J1** is poor selective for CE and **J2** has low response rate.

The probes **J4**‐**J7** (Figure 1C) are composed of different fatty ring‐structure trigger groups (from three‐membered ring to six‐membered ring). In kinetics studies, it was found that the probes (**J4** and **J5**) are hydrolyzed slowly to release the weak fluorescence majored at 676 nm within 1200 s (Figure 1E). **J6** and **J7** could not induce any response toward CE (Figure 1E), even after 2 h (Figure S39, Supporting Information). The inferior response kinetics suggested **J4**‐**J7** are not ideal probes for CE.

The probes **J8**‐**J12** and **J_Fast_** (Figure 1C) are composed of different aromatic ring‐structure trigger groups. Probes **J8**‐**J12** displayed slow response rate under physiological conditions (Figure 1F,G; Figure S40, Supporting Information). It is noted that *F*_676_ of **J_Fast_** increased at a rapid rate after addition of CE. Excitingly, **J_Fast_** only required 150 s to reach the maximum of fluorescent intensity, upon exaction at 630 nm. The ultrafast response rate is superior to the CE probes reported so far (Table S1, Supporting Information). Meanwhile, it was observed that **J_Fast_** also could avoid the interference of AChE and BChE. The addition of CE induced a 75‐fold fluorescence increase. While, **J_Fast_** had almost no any response to AChE and BChE (<3.7‐fold increase), which indicated that **J_Fast_** possess the combination with high selectivity and rapid response rate.

The above‐mentioned results indicated that different R moieties in amide group significantly affect the performance of probe to response CE, which may be related to a variety of factors, involving the affinity of probes to CE, distance from the probe to the active cavity of CE, etc. Thus, we employed molecular docking simulations to evaluate the potentials of the designed probes as CE substrates. The probes were well‐docked into the active cavity of human CES1 (PDB ID: 5A7H). The docking coefficient and distances from the probe to Ser221 (DisSer211) and His468 (DisHis468), the key serine and histidine residuals in the active cavity of human CES1,^[^ ^41^ ^]^ were obtained, as shown in Table S2 (Supporting Information). **J7**, **J11** and **J12** possess basically same DisSer211 value and spatial conformation in CE. However, the three probes displayed a clear difference in response rate, which may attribute to different binding coefficient. Compared to **J7** (60.04) and **J12** (58.39), **J11** possess a relatively lower binding coefficient with 56.01, favor bond‐breaking, which is comparable to rapid response dynamic of **J11**. The data indicated high binding affinity the residuals in the catalytic cavity of CE and probe may hinder the catalytic response, and affect the response rate of probe.

The binding coefficient of **J6** showed the same to that of **J_Fast_** (56.61). While, unlike **J_Fast_**, the fluorescence intensity of **J6** hardly changed after adding of CE (Figure 1F), which should be due to the longer DisSer211 (6.7 Å). **J_Fast_** presented shortest DisSer221 (4.0 Å) and DisHis468 (5.0 Å) in designed probes. Comparably, **J8** could also be docked into the active cavity of CE, but such the longer DisSer221 (7.1 Å) and DisHis468 (8.4 Å) compared to **J_Fast_**, which affect and hinder the process of CE‐mediated catalysis of **J8** (Figure 1G). Similar results were observed in **J9** and **J10** (Figure S41, Supporting Information). The introduction of heavy atoms (**J9**, DisSer221, 5.8 Å) and conjugated groups ((**J10**, DisSer221, 6.4 Å) increases the spatial distance between the probe and active cavity of CE, thus the response rate of **J9** and **J10** for CE decreases significantly, compared to **J_Fast_**.

### 2.2 Responsive Mechanism of J_Fast_

In order to confirm the responsive reaction mechanism of probes, reaction mixture containing **J_Fast_**, **J8**, **J6** and CE was incubated for 5 min and then subjected to HPLC analysis (Figure 1J). The peak intensity at *R*_t_ = 16.10 min (assigned to **J_Fast_**) was shown to have decreased significantly, together with concomitant increases of the peak (*R*_t_ = 21.38 min, belonging to **BB3**), after incubation with CE. And, the peak area of the reaction product (**BB3**) of **J_Fast_** and CE was more than that of **J8**. The co‐incubation of **J6** and CE did not induce the produce of peak of the reaction product (**BB3**). The data again demonstrated the excellent property of **J_Fast_** in detection of CE, which were comparable to fluorescence dynamic tests and computable simulation results.

### 2.3 Spectroscopic Properties of J_Fast_

**Figure 2:**
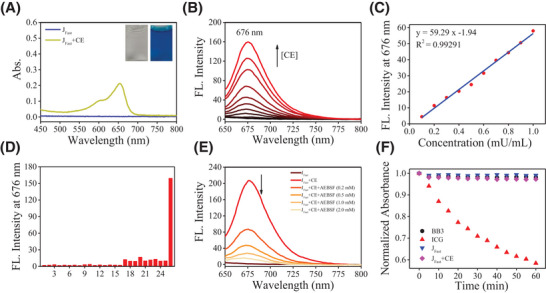
A) Absorption spectra of 5 µm **J_Fast_** upon addition of 10 U mL^−1^ CE. Inset of (A): the color change of **J_Fast_** in the absence and presence of CE. B) Fluorescent emission spectral change of 5 µm **J_Fast_** after addition of CE with 0, 0.1, 0.2, 0.4, 0.6, 0.8, 1.0, 2.0, 4.0, 6.0 mU mL^−1^ in 10 mm phosphate buffer (DMF 0.25%, v/v, pH = 7.4), upon excitation 630 nm, slit: 5 nm/5 nm. C) Detection limit of **J_Fast_** to CE. Plot of *F*_676_ of **J_Fast_** as a function of the CE concentration. D) Selectivity tests of **J_Fast_** to CE. *F*_676_ of 5 µm **J_Fast_** after addition of various analysis, upon excitation with 630 nm, respectively. 1 to 26 represent blank, 200 µm GSH, Hcy, Cys, 1 mm H_2_O_2_, CaCl_2_, KCl, MgCl_2_, Na_2_SO_3_, NaBr, NaCl, NaF, NaHSO_3_, NaNO_2_, NaOH, NaSCN, 50 µg mL^−1^ glutathione *S* transferase (GSTs), human serum albumin (HSA), lysozyme, ascorbic acid, 10 U mL^−1^ aminopeptidase N (APN), leucine aminopeptidase (LAP), *γ*‐glutamyl transferase (GGT), nitroreductase (NTR), NADPH Quinone Oxidoreductase 1 (NQO1) and CE, respectively. E) Inhibitory activity of **J_Fast_** against AEBSF with different concentrations. Fluorescence emission spectral change of 5 µm **J_Fast_** after addition of the mixture of 6 mU mL^−1^ CE and AEBSF with different concentrations (0.2, 0.5, 1.0, and 2.0 mm) for 40 min at 37 °C. F) Photostability of **BB3**, **ICG**, **J_Fast_** and the mixture of **J_Fast_** and CE in PBS detected via absorbance spectra. Every sample were continuously irradiated by laser with 640 nm (58 mW cm^−2^).

Above these results indicated that **J_Fast_** might be a good substrate for CE. Next, the detailed spectroscopic properties of **J_Fast_** were investigated in physiological conditions (DMF 0.25%, v/v, pH 7.4) to verify the validity of this probe. **J_Fast_** itself showed almost no absorbance peak from 450 nm to 800 nm. Upon reaction with CE, a new absorbance peak majored at 654 nm with a shoulder peak at 600 nm presented (**Figure** **2**A). After adding CE, **J_Fast_** presented a fluorescence turn‐on response centered at 676 nm, and the fluorescence intensity increased with the increase of CE concentration (Figure 2B). The plot between the concentration of CE and the emission intensity of **J_Fast_** is displayed, and good linearity was obtained at concentrations of 0–1.0 mU mL^−1^ with a correlation coefficient of 0.99291 (Figure 2C). The limit of detection of **J_Fast_** toward CE was estimated as 12.8 µU mL^−1^, which was comparable to those of the established probes (Table S1, Supporting Information). To investigate the interference, the reaction of the probe with various species was performed, including several inorganic salts, reactive oxygen species, reactive sulfur species or other enzymes (Figure 2D). However, only when treated with CE, the strongest and most distinct red fluorescence enhancement (75‐fold) was observed. These results indicate the super excellent selectivity and anti‐interference performance of **J_Fast_** toward other cellular species, which is required for selective and accurate detection under complex biosystems. Expectedly, a good linearity with a correlation coefficient of 0.99126 between the *F*_676_ and concentration of CE was also observed in cell lysate (Figure S42, Supporting Information). The effect of an inhibitor of CE, 4‐(2‐aminoethyl)benzenesulfonyl fluoride hydrochloride (AEBSF),^[^ ^29^ ^]^ was preincubated with CE, the fluorescence intensity of **J_Fast_** decreased, compared to the group (the mixture of **J_Fast_** and CE). In addition, with increasing concentration of AEBSF, the fluorescence intensity decreased dramatically (Figure 2E), indicating that the fluorescence change of the reaction indeed arose from CE‐catalytic hydrolysis. In addition, the photostability of **J_Fast_** was estimated based on time‐course absorbance measurements under continuous laser irradiation with 640 nm (58 mW cm^−2^) in an aqueous solution. NIR dye indocyanine green (ICG) was chosen as the control compound and displayed an almost 42% decrease after continuous exposure to a laser for 1 h (Figure 2F). However, < 3% absorbance fluctuation of **J_Fast_**, **BB3** and the mixture of **J_Fast_** and CE was calculated under the same conditions, indicating that high photo‐stabilities are highly desirable for tracking and bioimaging of CE activity in vivo.

### 2.4 Imaging CE Activity in Living Cells

**Figure 3:**
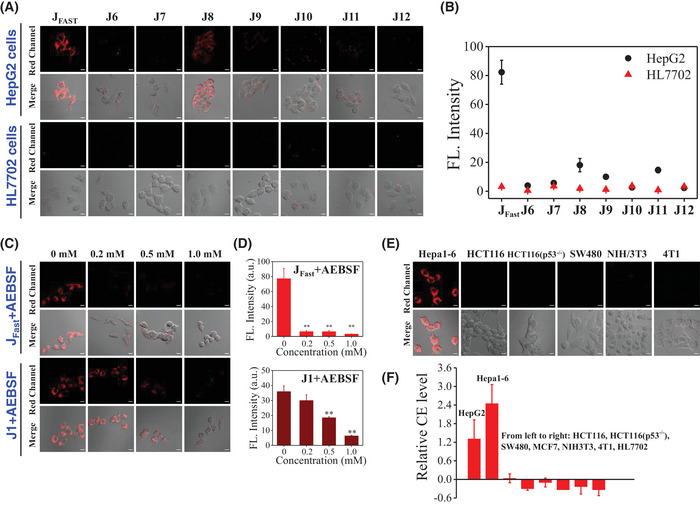
A) CLSM images of 10 µm probes‐loaded HepG2 or HL7702 cells for 30 min. B) Corresponding average fluorescence intensities of cells in red channels in (A); error bars are ± S.D. C) CLSM images of 10 µm **J_Fast_** or **J1**‐loaded HepG2 for additional stimulation with AEBSF under different concentrations (0, 0.2, 0.5, 1.0 mm) for 30 min. D) Corresponding average fluorescence intensities of cells in red channels in (C); **p < 0.01, error bars are ± S.D. E) CLSM images of 10 µm **J_Fast_**‐loaded Hepa1‐6, HCT116, HCT116(p53^−/−^), SW480, NIH/3T3 and 4T1 cells for 30 min. F) Analyses of CE levels by commercial kit in HepG2, Hepa1‐6, HCT116, HCT116/p53^−^, SW480, NIH/3T3, 4T1 and HL7702 cells. Red channel: 680±30 nm, *λ*_ex_ = 633 nm; scale bar = 10 µm.

We next assessed the property of these probes in living cells by using confocal laser scanning microscopy (CLSM). The HepG2 cell line (human liver cancer cell) and HL7702 cell line (human normal liver cell) were chosen as model cell lines to verify further the properties of probes (**Figure** **3**A,B). As reported, the activity of CE in HepG2 was relatively overexpressed, compared to HL7702 cells with low expressed levels.^[^ ^37^ ^]^ In CLSM experiments, HepG2 and HL7702 cells were incubated with 10 µm probes for 30 min. Then fluorescence signal was collected from the red fluorescence channel (680 ± 30 nm) with 633 nm as the excitation wavelength. After incubation with **J_Fast_** for 30 min, HepG2 cells showed bright red fluorescence, while almost no fluorescence could be observed in HL7702 cells (Figure 3A). **J_Fast_** also can be used to label specifically HepG2 cells in co‐culture model (HepG2 cells and HL7702 cells), as shown in Figure S43 (Supporting Information). HepG2 cells incubated with **J8** showed a slight increase in red fluorescence. Apart from that, there was no significant difference in signal intensity between HepG2 cells and HL7702 cells after incubation with other probes. Time‐dependent experiments revealed that fluorescence intensity of HepG2‐loaded with of **J_Fast_** and **J8** at red channel increased over time. After 30 min, 95‐fold fluorescence enhancement of **J_Fast_** (a 23‐fold increase of **J8**, Figure 3B) could be found, benefiting the rapid kinetics of **J_Fast_** to CE (Figure S44, Supporting Information). CLSM images of HepG2 cells co‐labeled with **J_Fast_**/Mitotracker or Lysotracker also were carried out in Figure S45 (Supporting Information). The merged images of the fluorescent intensities showed a weak colocalization of **J_Fast_** and Mitotracker or Lysotracker, indicating that **J_Fast_** was predominantly distributed in the whole cytoplasm.

To further investigate the imaging property of **J_Fast_**, an inhibitory experiment was implemented in HepG2 cells (Figure 3C,D). HepG2 cells loaded with **J_Fast_** were preincubated with AEBSF with different concentration for 30 min. The addition of the lowest dose (0.2 mm) of AEBSF directly induced a drastic decrease in red fluorescence intensity with a statistically significant (*p* < 0.01, Figure 3D), indicating that the fluorescence intensity indeed arose from the reaction of **J_Fast_** and CE. While, the addition of AEBSF with the same concentration could not completely inhibit the red fluorescence induced by **J1** (Figure 3F,G), which indicated the high selectivity of **J_Fast_** toward CE.

Moreover, Hepa1‐6 (mouse hepatoma cells), HCT116 (human colon cancer cells), HCT116(p53^−/−^) (p53 deficient HCT116 cells), SW480 (human colon cancer cells), NIH3T3 (mouse embryonic fibroblasts), 4T1 (mouse breast cancer cells) cell lines were employed to verify the high specificity of **J_Fast_** toward liver cancer cells. As shown in Figure 3E, after incubation with **J_Fast_**, under the same imaging condition, only hepatoma cells, involving Hepa1‐6 cells could be turned on and emitted bright red fluorescence. The analysis of CE level by a commercial kit further demonstrated that the CE level was higher in HepG2 cells and Hepa1‐6 cells than in other cells (Figure 3F), implying that CE is overexpressed in hepatoma cells, and **J_Fast_** is a potentially powerful tool to distinguish liver cancer cells from other cells.

Encouraged by the excellent performance of **J_Fast_** imaging and the overexpression of CE activity in HepG2 cells, we further evaluated the effect of the approved anti‐liver‐cancer drug (sorafenib)^[^ ^42^ ^]^ on CE activity. HepG2 and Hepa1‐6 cells loaded with **J_Fast_** for 30 min were pretreated with sorafenib for different times (0, 2, 4, 8, and 12 h). It was observed that the fluorescence intensity dramatically decreased (Figure S46, Supporting Information), indicating that the CE level also reduced, which is further **J_Fast_** has the capacity to monitor the change of CE activity with high selectivity and image the different conditions of liver cancer cells during the anticancer drug activation process.

In addition, a standard counting kit‐8 was employed to evaluate the potential toxicity and biocompatibility of **J_Fast_**. When incubated with 1–20 µm **J_Fast_** for 5 or 10 h in living HepG2 and Hepa1‐6 cells, the cell viabilities were more than 88%, indicating that probes possessed low acute toxicity and good biocompatibility (Figure S47, Supporting Information).

### 2.5 Imaging CE Activity in In Vivo

**Figure 4:**
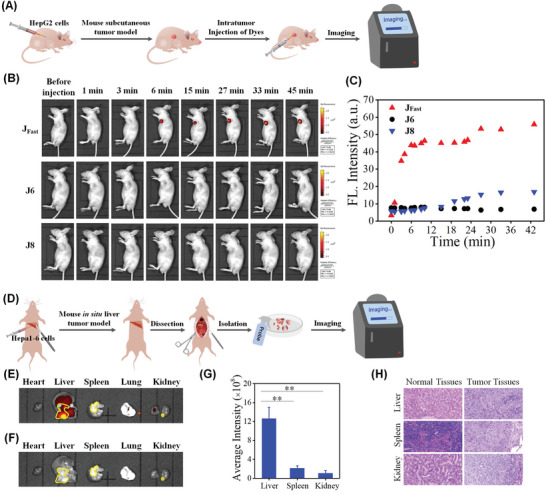
A) Schematic illustration of **J_Fast_**, **J6**, and **J8** imaging CE activity in subcutaneous HepG2‐xenografted tumor‐bearing mouse model. B) Time‐dependent in vivo imaging of CE activity in tumor‐bearing mice through intratumor injection of **J_Fast_**, **J6**, and **J8** (200 µm). C) Corresponding average fluorescence intensities of tumor region in red channels in (B). D) Schematic illustration of **J_Fast_** imaging CE activity in Hepa1‐6‐orthotopic tumor‐bearing mouse model. E) Fluorescent and F) brightfield imaging of CE in separated organs (heart, liver, spleen, lung, and kidney) sacrificed from model mice in (D). Yellow circles indicate the tumor region. G) Corresponding average fluorescence intensities of tumor region in liver, spleen, and kidney in yellow circles in (E), **p < 0.01, error bars are ± S.D. H) HE staining of normal and tumor tissues harvest from liver, spleen, and kidney harvest from (E). Red channel: 695–770 nm, *λ*_ex_ = 640 nm.

Motivated by the performance of **J_Fast_** in liver cancer cells, we monitored the property of this probe within the liver tumor model in in vivo (**Figure** **4**). **J_Fast_**, **J6** and **J8** were administered to the subcutaneous HepG2‐xenografted tumor‐bearing mouse model by intratumoral injection and scanned at different time points (0, 1, 3, 6, 15, 27, 33, and 45 min) using the vivo imaging system (Figure 4B). As shown in Figure 4C, noticeable red fluorescence enhancement could be observed after 6 min, indicating that **J_Fast_** could successfully image CE activity in the liver tumor in vivo. After injection with **J8**, the fluorescence intensity in tumor region presented undetectable increase, indicating the slow response kinetics of probe toward CE. While, under the same condition, tumor region **J6** injected did not exhibit observed fluorescence enhancement. These in vivo data demonstrated the rapid kinetics performance of **J_Fast_** for **CE**.

To further reflect the condition of natural liver cancer, an orthotopic liver cancer mice model was established through injection of Hepa1‐6 cell suspension to the left liver of the mice (Figure 4D). After ten days of feeding and raising, the mice were sacrificed and organs were collected and sprayed with a solution of **J_Fast_**. Brightfield images of separated organs displayed orthotopic liver tumor formation and simultaneously multiple organic metastasis (to spleen and kidney) appearance. In fluorescence imaging, bright red fluorescent signal was only collected in the liver (Figure 4E,F). In contrast, undetectable fluorescence was observed in the tumors of the spleen and kidney. The average fluorescence intensity in liver is significantly higher than that of spleen and kidney (**p < 0.01, Figure 4G), indicating that the probe bears targeting imaging to the orthotopic liver tumor. The HE staining results again indicated orthotopic tumor formation in liver, and metastatic tumor appearance in spleen and kidney (Figure 4H).

Next, we explored whether **J_Fast_** could indicate the therapeutic effect of sorafenib on the HepG2‐xenografted tumor‐bearing mouse model (Figure S48, Supporting Information). Noticeable fluorescence enhancement in 6 min could be observed after **J_Fast_** was administered to the mice model by intratumoral injection. While, the mice pretreated with sorafenib by intratumoral injection for a continuous one week, fluorescence intensity decreased at 6 min compared to that without sorafenib treatment, indicating that CE activity was inhibited by sorafenib.

### 2.6 Discriminating between Tumor Tissues and Normal Ones

**Figure 5:**
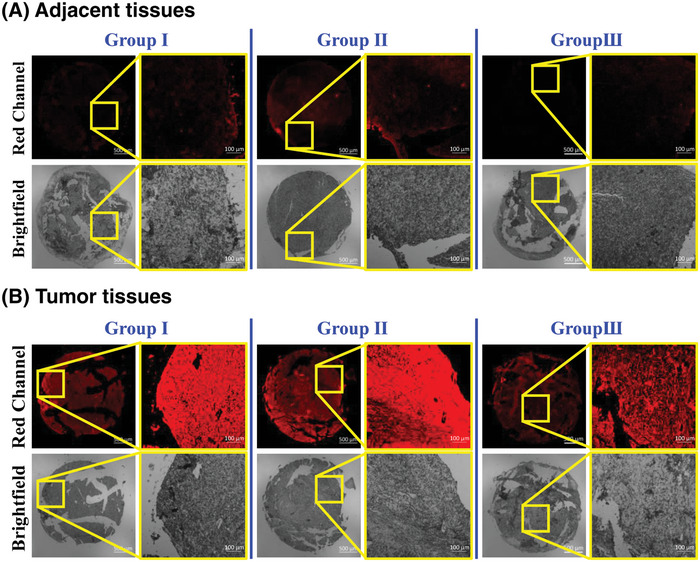
Confocal fluorescence images of A) adjacent and B) HCC tumor tissue sections (three groups) harvested from surgical specimens treated with **J_Fast_** (10 µm, 10 min). Right images are the amplified tissues in yellow squares of left images, in each group. Red channel: 680±30 nm, *λ*_ex_ = 633 nm.

Encouraged by the above results, we set out to evaluate the capability of **J_Fast_** to diagnose human orthotopic liver tumor tissues. In the assays, three group HCC tissues and three group adjacent specimens were incubated with **J_Fast_** for 10 min. The fluorescence image experiments were performed under CLSM. As shown in **Figure** **5**, three HCC tissue specimens showed bright fluorescence in the red channel (Figure 5B); in contrast, adjacent tissues presented faint red fluorescence (Figure 5A), which should be due to higher overexpression of CE in HCC tissues compared to that of adjacent tissues. This date indicated the great potential of **J_Fast_** in diagnosing HCC.

## 3 Conclusion

In summary, we have proposed a novel “substrate‐hydrolysis enzymatic reaction” strategy to construct a CE probe and employed an amide group as a CE‐specific recognition group. Under the guidance of this idea, a series of compounds were developed by combining basic blue 3 as fluorophore with different R‐substituted moieties at amide groups. The in vitro fluorescence kinetics and HPLC experimental data assisted by computational docking data were employed to investigate the effect of the nature of R‐substituted groups on the detection performance of the probes. An optimal NIR fluorescent probe (**J_Fast_**) was screened for monitoring CE activity with ultra‐rapid response rate (within 150 s) and super‐high selectivity (to avoid the interference of other esterase). The probe not only can distinguish HepG2 cells from other normal or cancer cells, but also can successfully be used to monitor the changes of CE activity in HepG2 cells treated with anti‐cancer drug (sorafenib) and in tumor‐bearing mice, giving it the potential to be used to assess the efficacy of anti‐hepatocellular carcinoma drugs in vivo. Notley, the probe was able to present significant fluorescence intensity differences between orthotopic liver tumor and normal organs or metastases in mice. Moreover, with the help of **J_Fast_**, a series of tumor tissue specimens harvested from patients have successfully been identified from adjacent tissues, indicating the great potential of the strategy for practical clinical applications of HCC diagnosis. The novel CE fluorescent probe will become an effective tool for in vivo and in situ monitoring of hepatocellular carcinoma status and assessing the efficacy of HCC drugs.

## Conflict of Interest

The authors declare no conflict of interest.

## Supporting information

Supporting Information

## Data Availability

Research data are not shared.
