# Supplementary material for: A Space‐Dependent ‘Enzyme‐Substrate’ Type Probe based on ‘Carboxylesterase‐Amide Group’ for Ultrafast Fluorescent Imaging Orthotopic Hepatocellular Carcinoma
Source: Adv Sci (Weinh). 2023 Jan 18;10(8):2206681. doi: 10.1002/advs.202206681 (PMC10015879; doi:10.1002/advs.202206681)
Supplement: Supplementary file 1 — Supporting Information [file ADVS-10-2206681-s001.pdf]

ADVANCED SCIENCE
Open Access

Supporting Information

for *Adv. Sci.*, DOI 10.1002/advs.202206681

A Space-Dependent 'Enzyme-Substrate' Type Probe based on 'Carboxylesterase-Amide Group' for Ultrafast Fluorescent Imaging Orthotopic Hepatocellular Carcinoma

*Ying Wen, Ning Jing, Min Zhang, Fangjun Huo, Zhuoyu Li and Caixia Yin**

Electronic Supporting Information

A space-dependent ‘enzyme-substrate’ type probe based on ‘carboxylesterase-amide’ for ultrafast fluorescent imaging orthotopic hepatocellular carcinoma

Ying Wen, [a] Ning Jing, [a] Min Zhang, [c] Fangjun Huo [b], Zhuoyu Li [d] and Caixia Yin [a]*

[a] Key Laboratory of Chemical Biology and Molecular Engineering of Ministry of Education, Key Laboratory of Materials for Energy Conversion and Storage of Shanxi Province, Institute of Molecular Science, Shanxi University, Taiyuan 030006, China.

[b] Research Institute of Applied Chemistry, Shanxi University, Taiyuan 030006, China.

[c] State Key Laboratory of Component-based Chinese Medicine, Tianjin University of Traditional Chinese Medicine, Tianjin 301617, China

[d] Institute of Biotechnology, Key Laboratory of Chemical Biology and Molecular Engineering of National Ministry of Education, Shanxi University, Taiyuan 030006, China

* E-mail: yincx@sxu.edu.cn

Contents

1. **Experimental Section** 3
2. **Summary of fluorescent probes for detection of CE** 5
3. **Synthesis and Characterization.** 8
   Synthesis details of J1. 8
   Synthesis details of J2. 8
   Synthesis details of J3. 9
   Synthesis details of J4. 9
   Synthesis details of J5. 9
   Synthesis details of J6. 9
   Synthesis details of J7. 9
   Synthesis details of J11. 9
   Synthesis details of J12. 9
   Synthesis details of J_{Fast}. 9
   Synthesis details of J8. 10
   Synthesis details of J9. 10
   Synthesis details of J10. 10
4. **Additional Fluorescence Tests** 29
5. **Additional Results derived from Molecular Docking Simulations** 30
6. **Additional Fluorescence Tests in Cell Lysate.** 32
7. **Additional Fluorescence Imaging in Living Cells** 32
8. **Cell Viability** 35
9. **Additional Fluorescence Imaging in *in vivo*** 35
10. **References** 36

1. Experimental Section

Materials. All chemicals and solvents were purchased as reagent grade and used without further purification unless otherwise noted. Esterase from porcine liver (#E3019-3.5KU), acetylcholinesterase from electrophorus electricus (AchE, #C3389-500UN) and butyrylcholinesterase from equine serum (BchE, #C7512-1.2KU) and other enzymes were purchased from Sigma-Aldrich. All enzyme stock solution was kept in -80°C or -20°C for keeping the activity. 4-(2-Aminoethyl)-benzenesulfonyl fluoride hydrochloride (AEBSF, an inhibitor for serine proteinase) and CCK-8 reagents were obtained from Beyotime Co., Ltd. Sorafenib (a drug that can treat hepatocellular carcinoma, # E0806540010) were purchased from Energy Chemical. Fetal bovine serum and penicillin/streptomycin were obtained from Biological Industries and Beijing Solarbio Science&Technoiogy Co., Ltd. respectively. Roswell Park Memorial Institute (RPMI-1640) and Dulbecco's Modified Eagle Medium (DMEM) were purchased from Beijing Solarbio Science&Technoiogy Co., Ltd. The CE activity assay kit was purchased from Nanjing Jiancheng Bioengineering Institute Co., Ltd.

Instruments. ^{1}H NMR and ^{13}C NMR spectra were recorded on the Bruker Avance-600 MHz and 151 MHz NMR spectrometers, respectively. ESI mass spectrometry was carried out on the AB Triple TOF 5600plus System. UV-visible spectra and steady-state emission experiments were performed on a Hitachi U-3900 spectrometer and a Hitachi F-7000 spectrometer, respectively. The cytotoxicity assay was measured by a Bio Tek ELX808 fully automated microplate reader. The cell imaging experiments were measured by Zeiss LSM880 Airyscan confocal laser scanning microscope. The *in vivo* imaging assays were performed in the Perkin Elmer Lumina LT small animal optical imaging system. The high-performance liquid chromatography (HPLC) experiments were performed on Shimadzu LC-20AT HPLC spectrometer.

Molecular docking simulations. Molecular docking was performed in this study with CDOCKER in Discovery Studio 2020 software. Before docking, the ligands were converted to 3D structure in SDF format, and prepared using the Prepare Ligands protocol to remove duplicates and generate dominant conformations. The crystal structure of human CES1 (PDB ID: 5A7H) was also prepared using the Prepare Protein with expurgations of waters and the original ligands. All the parameters in this program were set to the default values. The active cavity of human CES1 was defined as the binding site, with all other parameters as default. As the probe **J7** could not induce any response towards CES1, we concluded that the conformation of **J7** is quite anastomotic with the active cavity. Hence, other probes similar to conformation of **J7** were picked out. Based on these results, the -CDOCKER interaction values were obtained and the distances form the probe to Ser221 and His468 were measured.

Determination of the detection of limit. The detection limit was calculated based on the fluorescence titration of $\mathbf{J_{Fast}}$ in the presence of CE. The fluorescence intensity of probe was measured and standard deviation of the blank measurement was achieved. The detection limit was calculated by using detection limit with the following equation:

$$\text{Detection limit} = 3\sigma/k$$

Where σ is the standard deviation of the blank measurement, k is the slope between the fluorescence intensity versus various CE concentrations.

Photostability test. $\mathbf{J_{Fast}}$, $\mathbf{J_{Fast}}$+CE，**BB3** and ICG were dissolved in 1 mL PBS solution. Sample

tubes were exposed to 640 nm laser lamp (58 mW/cm^2) and kept some distance away from the lamp. A cold trap equipped with aqueous solution was placed between lamp and sample tubes. Absorbance spectra were measured of samples at intervals (5 min) for 1 h.

HPLC analysis. $\mathbf{J_{Fast}}$, **J6**, **J8**, **BB3**, CE, $\mathbf{J_{Fast}}$+CE, **J6**+CE and **J8**+CE (CE final concentration of 10 U/mL, other substances final concentration of 20 μM, all injection volumes of 10 μL, 1% DMSO aqueous solution, v/v) were characterized by Shimadzu LC-20AT HPLC spectrometer, respectively. It should be noted that the $\mathbf{J_{Fast}}$+CE, **J6**+CE and **J8**+CE groups need to be injected after 5 min of mixing to give sufficient enzyme reaction time.

Cell culture. Human liver carcinoma HepG2 cells, human mammary adenocarcinoma MCF-7 cell line, human normal liver HL7702 cell lines, human colorectal carcinoma SW480 cells, mouse embryonic fibroblast NIH/3T3 cells and mouse hepatocarcinoma Hepa1-6 cells were cultured and maintained in DMEM medium (supplemented with 10% fetal bovine serum and 1% penicillin/streptomycin) at 37 °C and 5% CO_2 in a humidified incubator. Human colorectal carcinoma HCT116 cells and HCT116(p53$^{-/-}$) cells were cultured in RPMI-1640 medium supplemented with 10% fetal bovine serum and 1% penicillin/streptomycin. HCT116(p53$^{-/-}$) cells were the gifted from Dr. XingKang Wu from Shanxi University [S1]. Other cell lines were provided by the Institute of Biochemistry and Cell Biology, SIBS, CAS (China).

Preparation of cell lysate and activity assay of CE in cell lysate. Strict operating procedures are performed according to the kit instructions.

Cytotoxicity assay. Cells Counting Kit-8 (CCK-8) assay was carried out to assess cytotoxicity of $\mathbf{J_{Fast}}$ against HepG2 and Hepa1-6 cells. Cells (10^4 cells/well) were plated into 96-well plates and then allowed to adhere for 24 h. Subsequently, the cells were incubated with 0, 5, 10, 15 and 20 µM of $\mathbf{J_{Fast}}$ at 37°C in an atmosphere of 5% CO_2 for 24 h. The CCK-8 solution (10 µL) was added to each well and incubated for 1 h, then the optical density (OD) was measured at 450 nm using a microplate reader. When the amount of the probe added was 0 µM, the cell viability value was set to 100%. The cell viability was calculated by use of equations shown below. Cell viability (%) = (Mean OD of sample × 100) / (Mean OD of the control group).

Confocal laser scanning microscope (CLSM) imaging. HepG2 cells, HL7702 cells, MCF-7 cells, SW480 cells, HCT116 cells, HCT116/p53 cells, NIH/3T3 cells and Hepa1-6 cells were cultured overnight 14 mm glass coverslips overnight and washed with PBS (3 times × 2 mL). After incubation with 10 μM probes for 30 min at 37°C, cells were subjected to CLSM imaging with an oil objective lens 63 ×. The red channel (680±30 nm) was used to collect the probes signal under 633 nm as the excitation wavelength. For inhibitors' experiments, HepG2 cells were pretreated with 0, 0.2, 0.5, 1.0 mM AEBSF. After 30 min, cells were treated with 10 μM probes for another 30 min. Before subjected to imaging, cells were washed with PBS (3 times × 2 mL). HepG2 cells and Hepa1-6 cells were pretreated with 10 μM sorafenib in PBS. After 0, 2, 4, 8, 12 h, cells were washed with PBS (3 times × 2 mL), and then treated with 10 μM probes. Before subjected to imaging, cells were washed with PBS (3 times × 2 mL). For time sequence experiments, HepG2 cells were treated with 10 μM probes for 2, 5, 10, 20, 30 min. Before subjected to imaging, cells were washed with PBS (3 times × 2 mL).

Fluorescence imaging of CE activity in a HepG2 subcutaneous tumor-bearing mouse model. All the animal experiments were performed by following the protocols approved by the Radiation Protection Institute of Drug Safety Evaluation Center in China (Production license: SYXK (Jin) 2018-0005). All animal experiments were performed according to the protocols approved by the Animal Ethics and Use Committee. Male BALB/c nude mice of 6-8 weeks old were purchased from

Beijing Viton River Experimental Technology Co., Ltd. The mice were housed under a 12-h light/dark cycle and were allowed free access to food and water. HepG2 cells (1.5 × 10^7 cells) were selected for transplantation into the axillae of mice. After fifteen days, tumor xenograft mice were given $\mathbf{J_{Fast}}$ (200 μM, 20 μL) by intratumoral injection during mouse anesthesia. Images were taken by a small animal live imaging system at different time points (0, 1, 3, 6, 15, 27, 33, and 45 min) upon excitation at 640 nm. The above experiments were performed on two other subcutaneous tumor-bearing mice after replacing $\mathbf{J_{Fast}}$ with **J6** and **J8**, respectively.

Fluorescence imaging in the orthotopic liver tumor model. Nude BABL/c mice were anesthetized and opened via midline incision to expose the liver. Hepa1-6 cell suspension (1.5 × 10^7 cells) was slowly injected the left liver lobe of the mice, and the treated live was send back to the abdominal cavity. After 10 days of tumor formation in culture, the mice were dissected and the major organs (heart, liver, spleen, lung, and kidney) and tumor parts were removed, and the removed organs were sprayed with 200 μM of $\mathbf{J_{Fast}}$ solution and then fluorescent imaging using a small animal live imaging system.

Tissue slice imaging of human hepatocellular carcinoma. All tissue slice samples from 6 patients (adjacent tissues, n = 3; tumor tissues, n = 3) were harvested from Shanghai outdo biotech co.,ltd. Data and samples were prospectively collected with local ethics committee approval at each center, and all patients gave their informed consent to participate in the research. Adjacent tissues were identified for longitudinal assessment of liver function. The imaging of CE in the tissue slice samples was carried out using the designed $\mathbf{J_{Fast}}$ probe, and imaged in CLSM.

2. Summary of fluorescent probes for detection of CE

Table S1. Summary of fluorescent probes for detection of CE

Structures	$\lambda_{ex}/\lambda_{em}$ (nm)	Fluorescence Spectral Change	Testing Condition	Detection Limit	Bioimaging Application	Ref.
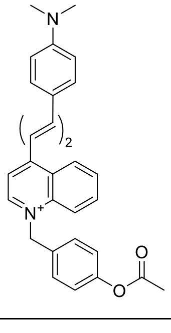	443/593	593↑<br>10 μM probe<br>8 U/mL CE	37°C, 40 min (PBS buffer, pH 7.4, 1% DMSO)	3.04 μU/mL	Beas-2B cells, L02 cells, MCF-7 cells, HeLa cells, HepG2 cells, tumor-bearing mice	S2
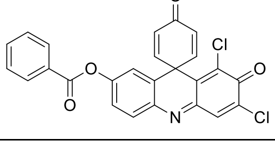	630/678	678↑<br>10 μM probe<br>10 μg/mL CES2	37°C, 30 min (100 mM PBS buffer, pH 7.4)	0.03 μg/mL	HepG2 cells	S3
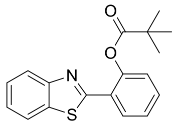	325/460	460↑<br>10 μM probe<br>10 μg/mL CE	30°C, 20 min (10 mM PBS buffer, pH 7.4)	40.2 μU/mL	HepG2 cells, A549 cells, $SKOV_3$ cells, HeLa cells,	S4

					zebrafish, living mice	
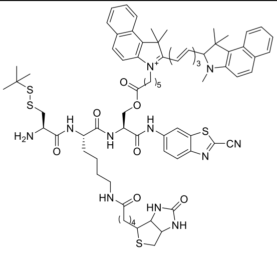	685/720	720↑<br>10 μM probe<br>0.1 nmol/U CE	37°C, 6 h (10 mM PBS buffer, pH 7.4, 10% DMSO)	-	HepG2 cells, L02 cells, tumor-bearing mice	S5
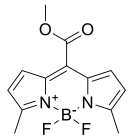	505/560	560↑<br>10 μM probe<br>1 μg/mL CES1	37°C, 30 min (PBS buffer, pH 7.4)	-	A549 cells, zebrafish	S6
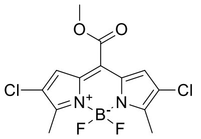	530/595	595↑<br>5 μM probe<br>1 μg/mL CES1	37°C, 30 min (100 mM PBS buffer, pH 7.4, ≤1% DMSO)	-	Caco-2 cells, liver slice of mice, zebrafish	S7
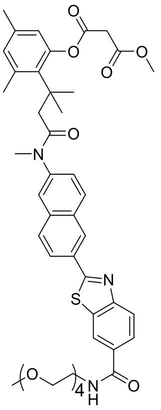	OP: 373/455, 540<br>TP: 740/400-450, 500-600	455↓, 540↑<br>1 μM probe<br>10 μg/mL CES2	37°C, 10 min (10 mM PBS buffer, pH 7.4)	-	HCT 116 cells, MDA-MB 468 cells, MDA-MB 231 cells	S8
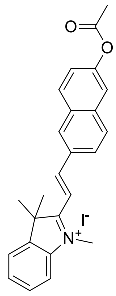	OP: 443/567<br>TP: 820/567	567↑<br>10 μM probe<br>0.1 U/mL CE	37°C, 7 min (5 mM PBS buffer, pH 7.4)	1.8 U/L	HeLa cells,	S9

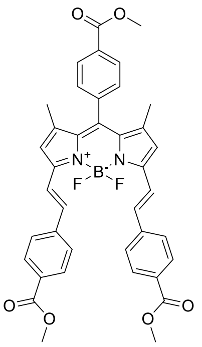	575/649	649↑ 10 μM probe 5 μg/mL CES1	37°C, 30 min (CH_3CN/PBS buffer, v/v, 1/1)	2.5 ng/mL	HepG2 cells	S10
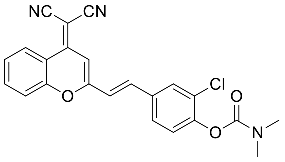	540/685	685↑ 5 μM probe 1 U/mL CE	37°C, 5 h (PBS buffer, pH 7.4)	13 mU/L	HepG2 cells, liver tumor tissue, living mice	S11
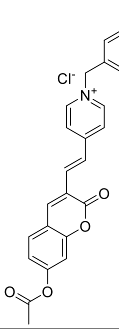	OP: 410/550 TP: 810/550	OP/TP: 550↑ 10 μM probe 15 μg/mL CE	37°C, 10 min (40 mM B-R buffer, pH 7.42)	0.12 μg/mL	HepG2 cells, liver of mice	S12
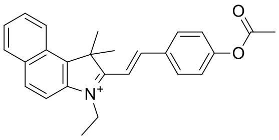	520/575	575↑ 10 μM probe 5 U/mL CE	37°C, 20 min (PBS buffer, pH 7.4)	0.12 mU/mL	A549 cells, HepG2 cells, mice liver slices	S13
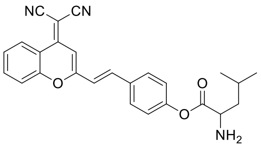	OP: 560/685 TP: 830/685	685↑ 10 μM probe 5 μg/mL CES2	37°C, 10 min (10 mM PBS buffer, pH 7.4)		HT-29 cells, slice of colonic cancer tissues, tumor-bearing mice	S14
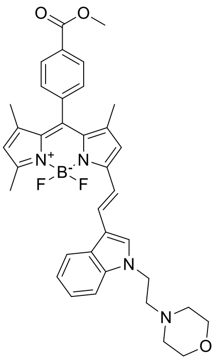	595/638	638↓ 10 μM probe 10 μg/mL CES1	37°C, 60 min (100 mM PBS buffer, pH 7.4)	0.087 μg/mL	HepG2 cells	S15

3. Synthesis and Characterization.

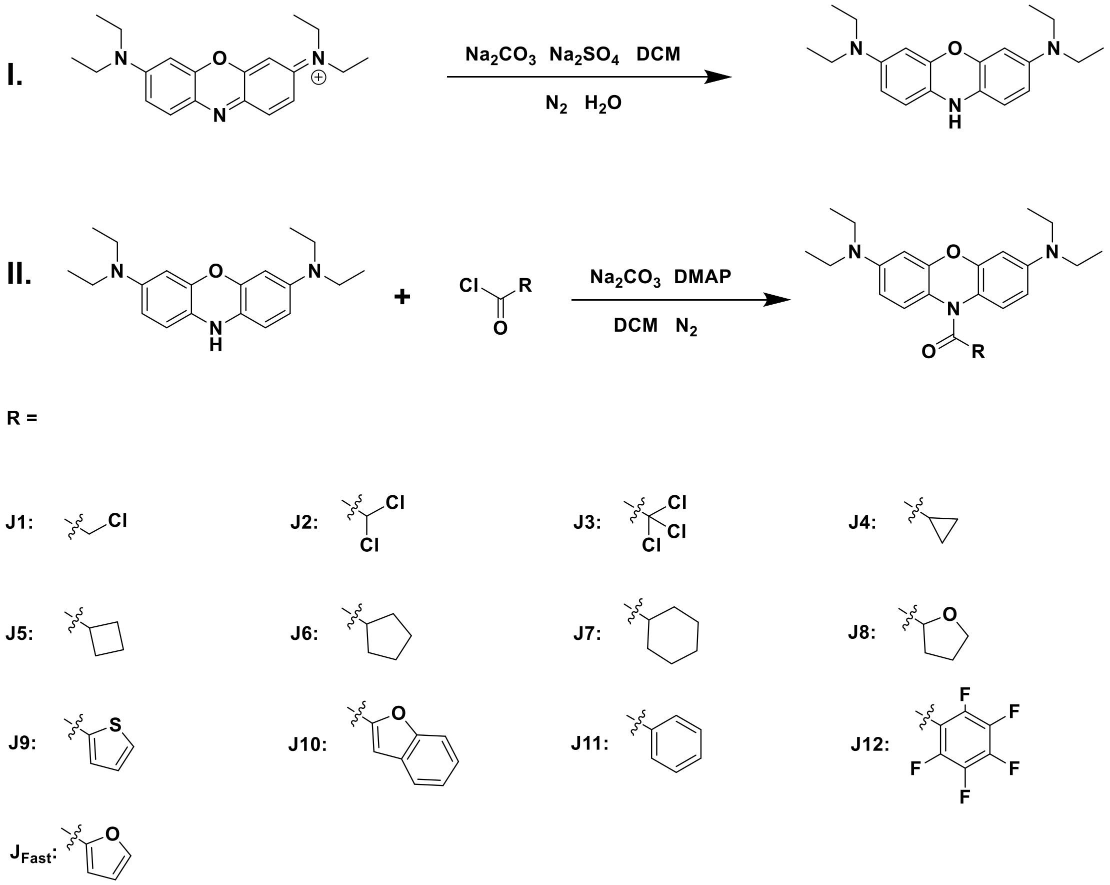

Scheme S1. Synthetic route of **J1**-**J12** and **J_{Fast}**.

Synthesis details of J1. I Basic Blue 3 (5 mmol, 1795 mg) and sodium carbonate (20 mmol, 2120 mg) were dissolved in 30 mL DCM. The mixture was stirred at 45℃ under the protection with nitrogen. After 5 min, sodium hyposulfite (20 mmol, 3482 mg) dissolved in 20 mL water was slowly added, and then stirred for additional 30 min. The organic phase was isolated in an anaerobic environment and immediately proceeded to the next reaction step.

II 4-Dimethylaminopyridine (5 mmol, 610mg) and sodium carbonate (12.5 mmol, 1325 mg) were added in the organic phase in reaction **I.** The mixture was stirred in an ice bath under the protection with nitrogen. After then, chloroacetyl chloride (6 mmol, 678 mg) was added slowly. After 2 h, the crude product was filtered and purified by column chromatography to give compound **J1** with petroleum ether/ethyl acetate = 6:1[S16-18]. **J1** (pale yellow powder, 1.25 g, yield 62.32%). ^{1}H NMR (600 MHz, DMSO) δ 7.36 (d, J = 8.8 Hz, 2H), 6.45 – 6.41 (m, 2H), 6.41 (s, 2H), 4.57 (s, 2H), 3.32 (d, J = 6.9 Hz, 8H), 1.08 (t, J = 6.9 Hz, 12H). ^{13}C NMR (151 MHz, $CDCl_3$) δ 165.26, 152.02, 147.28, 124.43, 117.38, 106.38, 99.68, 44.59, 41.74, 12.51. HR-MS: Calc. for $C_{22}H_{29}N_3O_2Cl^+$ $[M+H]^+$ 402.1948, found 402.1938.

Synthesis details of J2. J2 was synthesized as the synthetic methods of **J1**. **J2** (white powder 1.40 g, yield 64.35%). ^{1}H NMR (600 MHz, DMSO) δ 7.39 (s, 2H), 7.02 (s, 1H), 6.49 – 6.41 (m, 4H), 3.36 – 3.33 (m, 8H), 1.09 (t, J = 7.0 Hz, 12H). ^{13}C NMR (151 MHz, DMSO) δ 161.93, 151.67, 147.11, 124.44,

116.14, 106.39, 98.97, 65.90, 43.77, 12.23. HR-MS: Calc. for $C_{22}H_{28}N_3O_2Cl_2^+$ $[M+H]^+$ 436.1559, found 436.1553.

Synthesis details of J3. J3 was synthesized as the synthetic methods of **J1**. **J3** (light blue powder 1.36 g, yield 57.98%). ^{1}H NMR (600 MHz, $CDCl_3$) δ 7.39 (s, 2H), 6.39 (s, 4H), 3.34 (d, J = 5.8 Hz, 8H), 1.17 (s, 12H). HR-MS: Calc. for $C_{22}H_{27}N_3O_2Cl_3^+$ $[M+H]^+$ 470.1169, found 470.1162.

Synthesis details of J4. J4 was synthesized as the synthetic methods of **J1**. **J4** (white powder 1.20 g, yield 61.03%). ^{1}H NMR (600 MHz, $CDCl_3$) δ 7.42 (d, J = 7.9 Hz, 2H), 6.42 – 6.35 (m, 4H), 3.33 (q, J = 7.1 Hz, 8H), 2.16 – 2.10 (m, 1H), 1.57 (d, J = 10.0 Hz, 2H), 1.15 (t, J = 7.1 Hz, 12H), 0.80 (td, J = 6.6, 3.5 Hz, 2H). ^{13}C NMR (151 MHz, $CDCl_3$) δ 172.27 (s), 151.99 (s), 146.72 (s), 125.21 (s), 118.55 (s), 106.31 (s), 99.87 (s), 44.60 (s), 12.63 (s), 12.55 (s), 9.16 (s). HR-MS: Calc. for $C_{24}H_{32}N_3O_2^+$ $[M+H]^+$ 394.2495, found 394.2495.

Synthesis details of J5. J5 was synthesized as the synthetic methods of **J1**. **J5** (white powder 1.32 g, yield 64.82%).^{1}H NMR (600 MHz, DMSO) δ 7.24 (s, 2H), 6.41 (d, J = 8.7 Hz, 2H), 6.38 (s, 2H), 3.67 – 3.60 (m, 1H), 3.33 (d, J = 6.3 Hz, 8H), 2.17 – 2.09 (m, 2H), 1.90 (d, J = 9.0 Hz, 2H), 1.81 (dd, J = 18.6, 9.6 Hz, 1H), 1.70 (d, J = 9.9 Hz, 1H), 1.08 (t, J = 7.0 Hz, 12H). ^{1}H NMR (600 MHz, $CDCl_3$) δ 6.41 – 6.36 (m, 4H), 3.63 (s, 1H), 3.33 (q, J = 7.0 Hz, 8H), 2.40 (dd, J = 11.6, 8.9 Hz, 2H), 2.01 (d, J = 7.8 Hz, 2H), 1.88 (dd, J = 11.1, 6.7 Hz, 2H), 1.15 (t, J = 7.0 Hz, 12H). ^{13}C NMR (151 MHz, $CDCl_3$) δ 174.20 (s), 152.09 (s), 146.79 (s), 124.80 (s), 118.45 (s), 106.37 (s), 99.80 (s), 44.62 (s), 37.35 (s), 25.74 (s), 17.89 (s), 12.52 (s). HR-MS: Calc. for $C_{25}H_{34}N_3O_2^+$ $[M+H]^+$ 408.2651, found 408.2650.

Synthesis details of J6. J6 was synthesized as the synthetic methods of **J1**. **J6** (white powder 1.25 g, yield 59.34%). ^{1}H NMR (600 MHz, DMSO) δ 7.27 (d, J = 8.7 Hz, 2H), 6.41 (d, J = 8.9 Hz, 2H), 6.39 (s, 2H), 3.36 – 3.31 (m, 8H), 3.28 (d, J = 8.0 Hz, 1H), 1.66 (d, J = 18.6 Hz, 4H), 1.64 – 1.58 (m, 2H), 1.50 – 1.43 (m, 2H), 1.08 (t, J = 7.0 Hz, 12H). ^{13}C NMR (151 MHz, $CDCl_3$) δ 176.39 (s), 152.44 (s), 146.77 (s), 125.15 (s), 118.70 (s), 106.29 (s), 99.78 (s), 44.59 (s), 41.12 (s), 31.53 (s), 26.41 (s), 12.55 (s). HR-MS: Calc. for $C_{26}H_{36}N_3O_2^+$ $[M+H]^+$ 422.2808, found 422.2808.

Synthesis details of J7. J7 was synthesized as the synthetic methods of **J1**. **J7** (white powder 1.42 g, yield 65.24%). ^{1}H NMR (600 MHz, DMSO) δ 7.25 (d, J = 8.4 Hz, 2H), 6.42 (d, J = 8.7 Hz, 2H), 6.39 (s, 2H), 2.91 (t, J = 11.3 Hz, 1H), 1.67 (d, J = 9.9 Hz, 4H), 1.58 (s, 1H), 1.41 – 1.30 (m, 2H), 1.14 (dd, J = 21.4, 6.5 Hz, 3H), 1.08 (t, J = 6.8 Hz, 12H). ^{13}C NMR (151 MHz, $CDCl_3$) δ 175.63, 152.49, 146.78, 125.10, 118.53, 106.30, 99.83, 44.58, 40.59, 29.90, 25.77, 25.66, 12.55. HR-MS: Calc. for $C_{27}H_{38}N_3O_2^+$ $[M+H]^+$ 436.2964, found 436.2960.

Synthesis details of J11. J11 was synthesized as the synthetic methods of **J1. J11** (yellow powder 1.36 g, yield 63.37%).^{1}H NMR of **J11**. ^{1}H NMR (600 MHz, DMSO) δ 7.39 (dd, J = 8.8, 4.7 Hz, 1H), 7.33 (s, 2H), 7.33 (s, 2H), 7.06 (d, J = 7.9 Hz, 2H), 6.42 (d, J = 2.5 Hz, 2H), 6.24 (dd, J = 8.9, 2.2 Hz, 2H), 3.30 (q, J = 6.8 Hz, 8H), 1.06 (t, J = 7.0 Hz, 12H) ^{13}C NMR (151 MHz, $CDCl_3$) δ 167.61, 151.55, 146.72, 136.38, 129.86, 129.00, 127.97, 125.23, 119.02, 106.44, 99.80, 44.70, 12.65. HR-MS: Calc. for $C_{27}H_{32}N_3O_2^+$ $[M+H]^+$ 430.2495, found 430.2490.

Synthesis details of J12. J12 was synthesized as the synthetic methods of **J1**. **J12** (white powder 1.33 g, yield 51.23%). ^{1}H NMR (600 MHz, DMSO) δ 7.64 (d, J = 8.1 Hz, 1H), 6.50 (dd, J = 26.6, 7.7 Hz, 2H), 6.44 (d, J = 14.9 Hz, 2H), 6.14 (d, J = 7.4 Hz, 1H), 3.36 (s, 8H), 1.06 (d, J = 43.9 Hz, 12H). ^{19}F NMR (376 MHz, DMSO-d_6) δ -141.03 – -141.59 (m, 2F), -151.83 (t, J = 23.3 Hz, 1F), -160.08 – -160.91 (m, 2F). HR-MS: Calc. for $C_{27}H_{27}FN_3O_2^+$ $[M+H]^+$ 520.2023, found 520.2023.

Synthesis details of J_{Fast}. J_{Fast} was synthesized as the synthetic methods of **J1**. **J_{Fast}** (yellow powder 1.62 g, yield 77.29%). ^{1}H NMR (600 MHz, DMSO) δ 7.74 (d, J = 0.9 Hz, 1H), 7.23 (d, J = 9.0 Hz, 2H),

6.53 (dd, J = 3.5, 1.7 Hz, 1H), 6.51 (d, J = 3.4 Hz, 1H), 6.43 (d, J = 2.7 Hz, 2H), 6.34 (dd, J = 9.0, 2.7 Hz, 2H), 3.33 – 3.29 (m, 8H), 1.08 (t, J = 7.0 Hz, 12H). ^{13}C NMR (151 MHz, DMSO) δ 157.05 (s), 151.42 (s), 147.31 (s), 147.01 (s), 145.51 (s), 124.74 (s), 118.13 (s), 116.77 (s), 111.84 (s), 106.63 (s), 99.53 (s), 44.28 (s), 40.42 (d, J = 20.9 Hz), 40.22 (s), 40.22 (s), 40.08 (s), 39.94 (s), 39.80 (s), 39.66 (s), 12.76 (s). HR-MS: Calc. for $C_{25}H_{30}N_3O_3^+$ [M+H]$^+$ 420.2287, found 420.2284.

Synthesis details of J8. J8 was synthesized as the synthetic methods of **J1**. **J8** (yellow powder 1.21 g, yield 57.18%). ^{1}H NMR (600 MHz, $CDCl_3$) δ 7.46 (s, 2H), 6.40 – 6.36 (m, 4H), 4.97 (dd, J = 7.3, 5.3 Hz, 1H), 4.09 (d, J = 7.3 Hz, 1H), 3.93 (dd, J = 10.2, 5.0 Hz, 1H), 3.33 (q, J = 7.0 Hz, 8H), 2.20 – 2.08 (m, 2H), 1.87 (ddd, J = 14.4, 13.1, 7.5 Hz, 2H), 1.15 (t, J = 7.0 Hz, 12H). ^{13}C NMR (151 MHz, $CDCl_3$) δ 165.11 (s), 146.22 (s), 141.01 (s), 119.18 (s), 112.13 (s), 100.63 (s), 93.95 (s), 68.99 (s), 63.65 (s), 38.83 (s), 23.56 (s), 20.31 (s), 6.66 (s). HR-MS: Calc. for $C_{25}H_{34}N_3O_3^+$ [M+H]$^+$ 424.2600, found 424.2595.

Synthesis details of J9. J9 was synthesized as the synthetic methods of **J1**. **J9** (yellow powder 1.25 g, yield 57.45%). ^{1}H NMR (600 MHz, $CDCl_3$) δ 7.39 – 7.36 (m, 1H), 7.32 (d, J = 8.9 Hz, 2H), 7.07 – 7.04 (m, 1H), 6.87 (dd, J = 4.7, 4.0 Hz, 1H), 6.43 (d, J = 2.2 Hz, 2H), 6.29 (dd, J = 8.9, 2.3 Hz, 2H), 3.33 (q, J = 7.0 Hz, 8H), 1.15 (t, J = 7.1 Hz, 12H). ^{13}C NMR (151 MHz, $CDCl_3$) δ 161.04, 151.93, 147.02, 138.46, 131.95, 130.02, 126.59, 125.38, 118.83, 106.44, 99.68, 44.71, 12.63. HR-MS: Calc. for $C_{25}H_{30}N_3O_2S^+$ [M+H]$^+$ 436.2059, found 436.2055.

Synthesis details of J10. J10 was synthesized as the synthetic methods of **J1**. **J10** (yellow powder 1.32 g, yield 56.26%). ^{1}H NMR (600 MHz, $CDCl_3$) δ 7.55 (d, J = 7.8 Hz, 1H), 7.45 (d, J = 8.3 Hz, 1H), 7.35 (dd, J = 16.7, 9.2 Hz, 3H), 7.23 (t, J = 7.5 Hz, 1H), 6.81 (s, 1H), 6.50 (s, 2H), 6.31 (d, J = 8.1 Hz, 2H), 3.34 (q, J = 7.0 Hz, 8H), 1.16 (t, J = 7.0 Hz, 12H). ^{13}C NMR (151 MHz, $CDCl_3$) δ 157.84, 154.79, 151.60, 148.80, 147.04, 127.25, 126.51, 124.77, 123.42, 122.46, 118.50, 112.19, 112.08, 106.69, 99.92, 44.86, 12.62. HR-MS: Calc. for $C_{29}H_{32}N_3O_3^+$ [M+H]$^+$ 470.2444, found 470.2359.

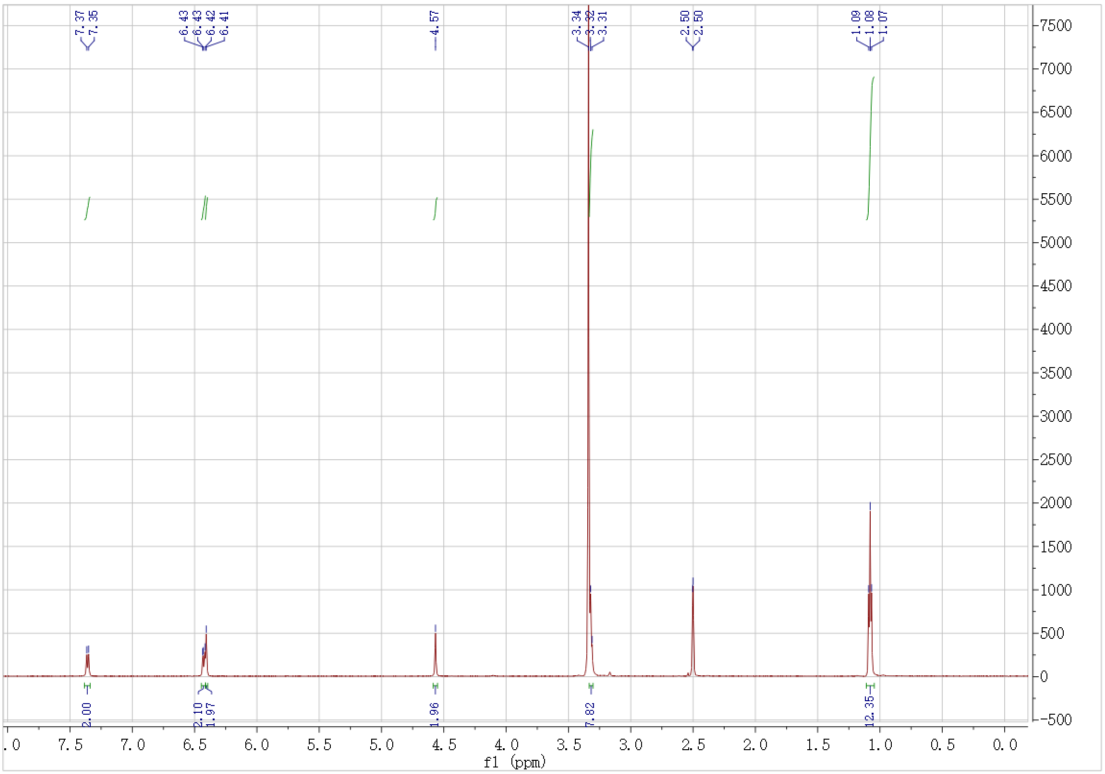

Figure S1. ^{1}H NMR spectrum of **J1** in DMSO-d_6. ^{1}H NMR (600 MHz, DMSO) δ 7.36 (d, J = 8.8 Hz,

2H), 6.45 – 6.41 (m, 2H), 6.41 (s, 2H), 4.57 (s, 2H), 3.32 (d, J = 6.9 Hz, 8H), 1.08 (t, J = 6.9 Hz, 12H).

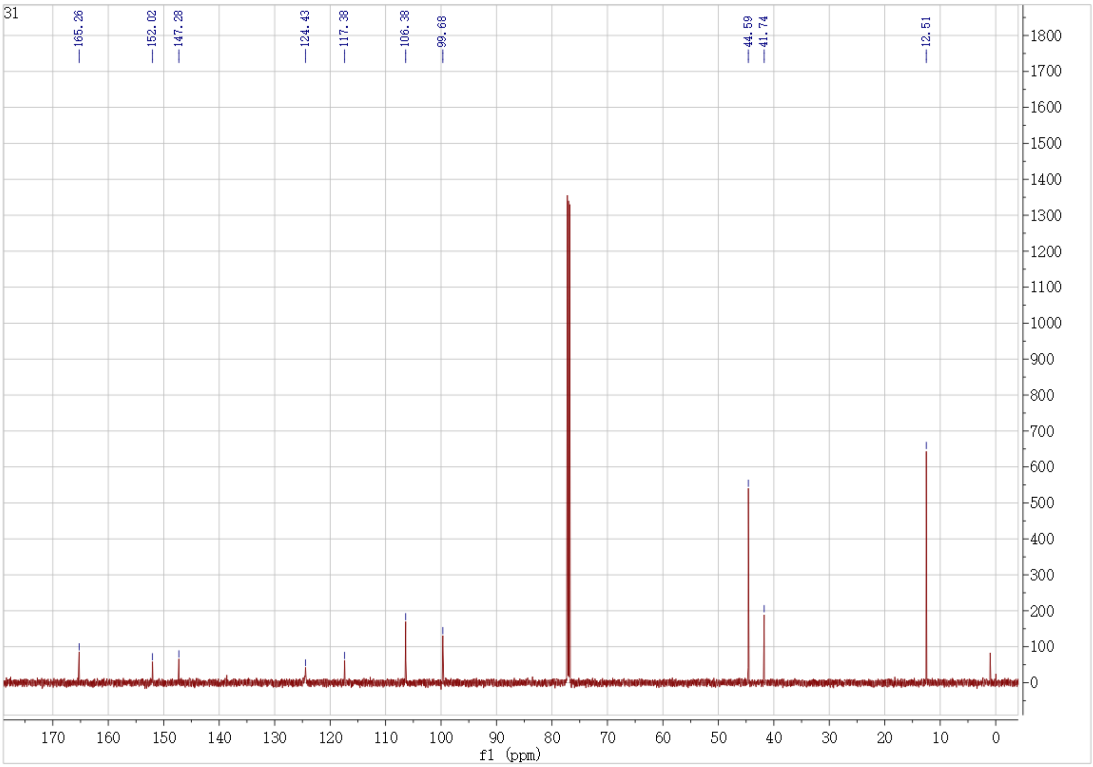

Figure S2. ^{13}C NMR spectrum of **J1** in $CDCl_3$. ^{13}C NMR (151 MHz, $CDCl_3$) δ 165.26, 152.02, 147.28, 124.43, 117.38, 106.38, 99.68, 44.59, 41.74, 12.51.

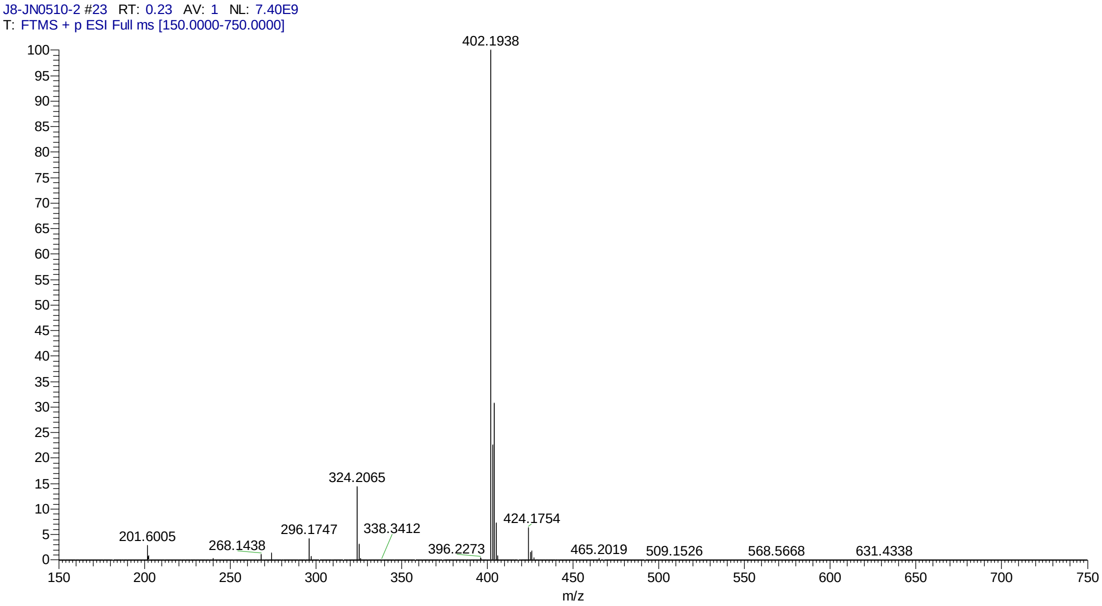

Figure S3. HR-MS of **J1**. Calc. for $C_{22}H_{29}N_3O_2Cl^+$ $[M+H]^+$ 402.1948, found 402.1938.

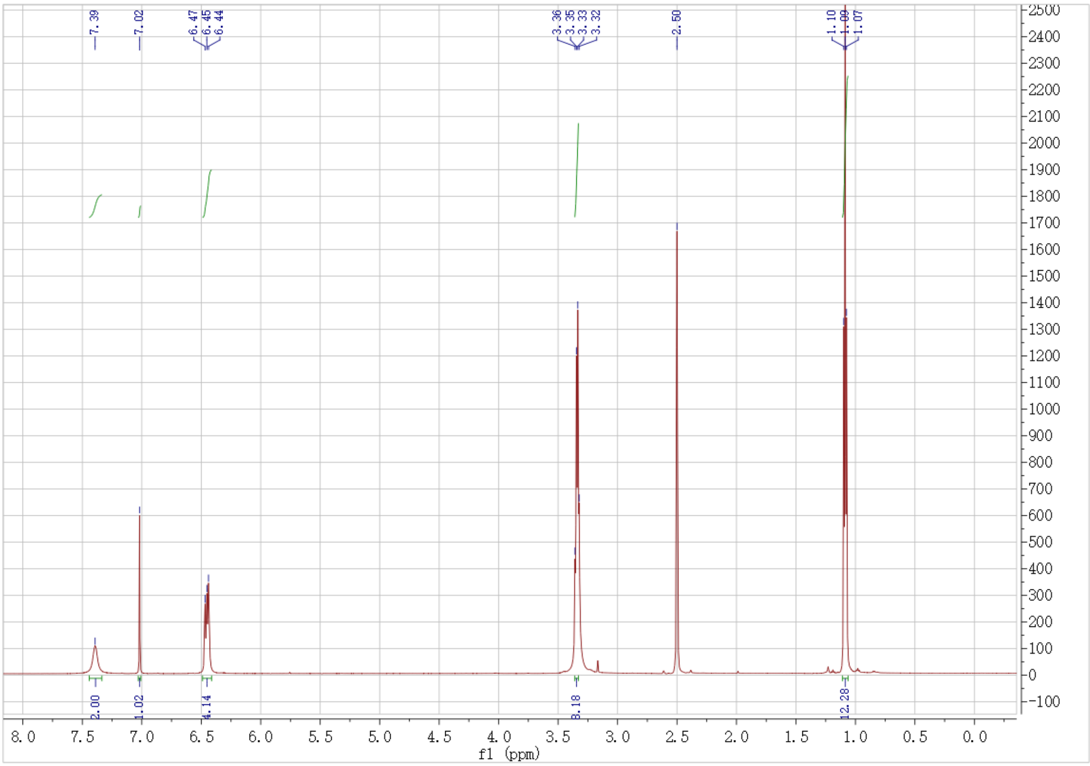

Figure S4. ^{1}H NMR spectrum of **J2** in DMSO-d_6. ^{1}H NMR (600 MHz, DMSO) δ 7.39 (s, 2H), 7.02 (s, 1H), 6.49 – 6.41 (m, 4H), 3.36 – 3.33 (m, 8H), 1.09 (t, J = 7.0 Hz, 12H).

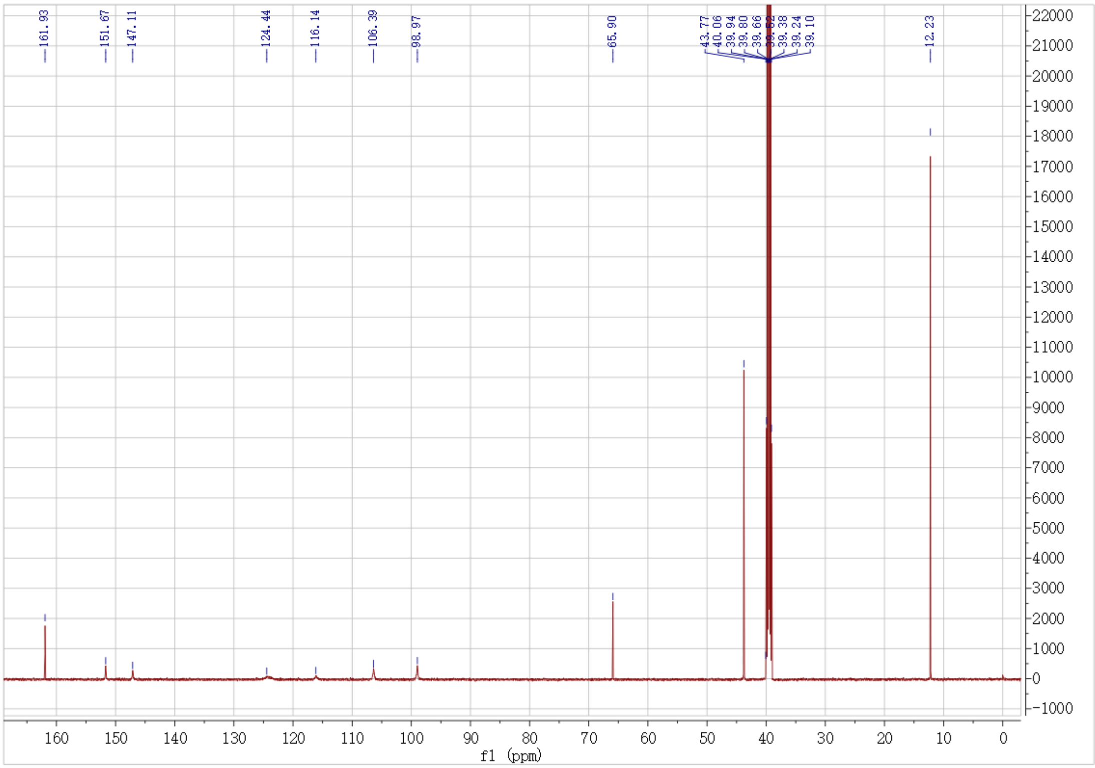

Figure S5. ^{13}C NMR spectrum of **J2** in DMSO-d_6. ^{13}C NMR (151 MHz, DMSO) δ 161.93, 151.67, 147.11, 124.44, 116.14, 106.39, 98.97, 65.90, 43.77, 12.23.

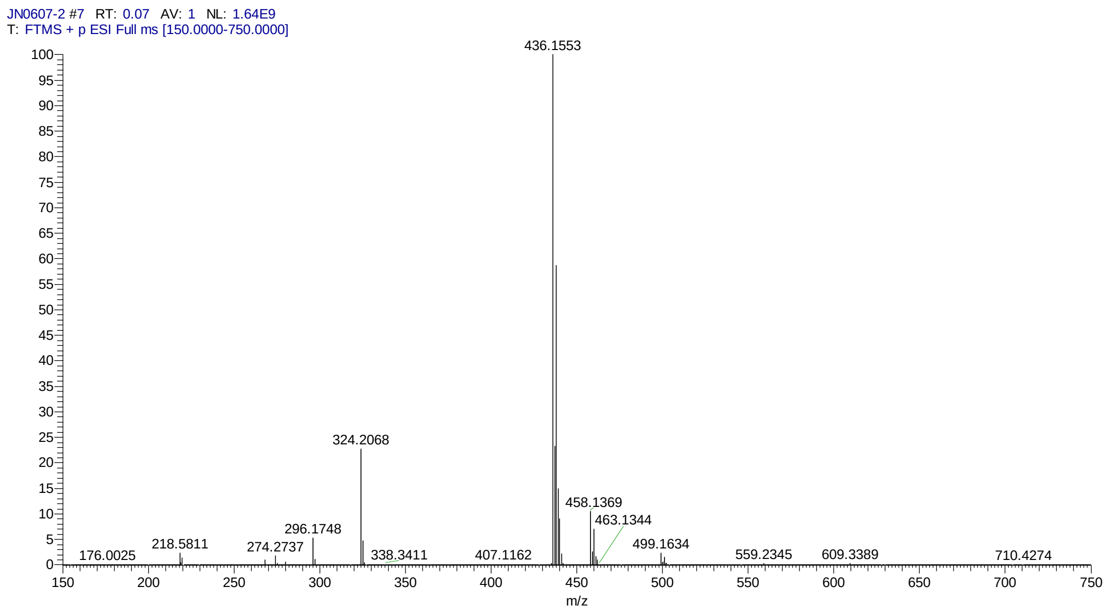

Figure S6. HR-MS of **J2**. Calc. for $C_{22}H_{28}N_3O_2Cl_2^+$ $[M+H]^+$ 436.1559, found 436.1553.

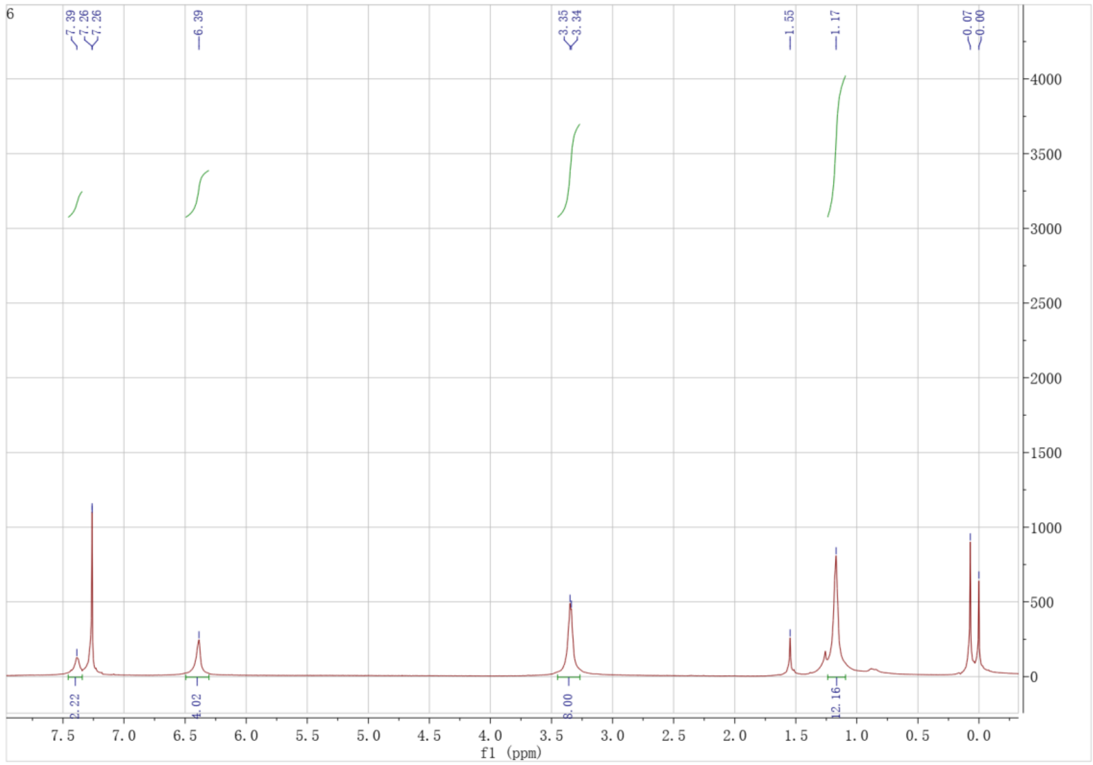

Figure S7. ^{1}H NMR spectrum of **J3** in DMSO-d_6. ^{1}H NMR (600 MHz, $CDCl_3$) δ 7.39 (s, 2H), 6.39 (s, 4H), 3.34 (d, J = 5.8 Hz, 8H), 1.17 (s, 12H).

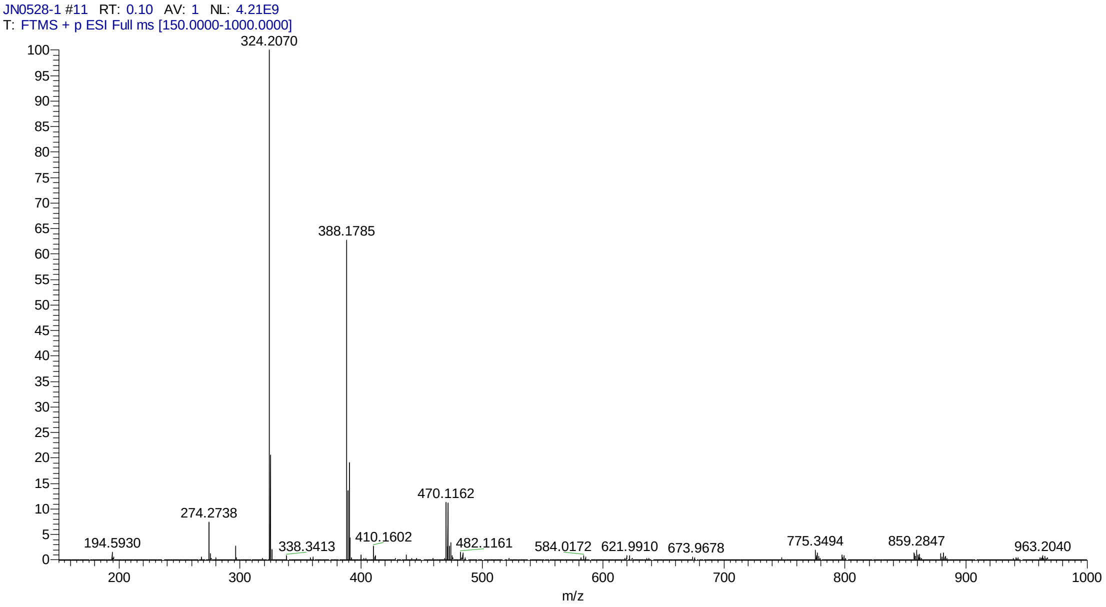

Figure S8. HR-MS of **J3**. Calc. for $C_{22}H_{27}N_3O_2Cl_3^+$ $[M+H]^+$ 470.1169, found 470.1162.

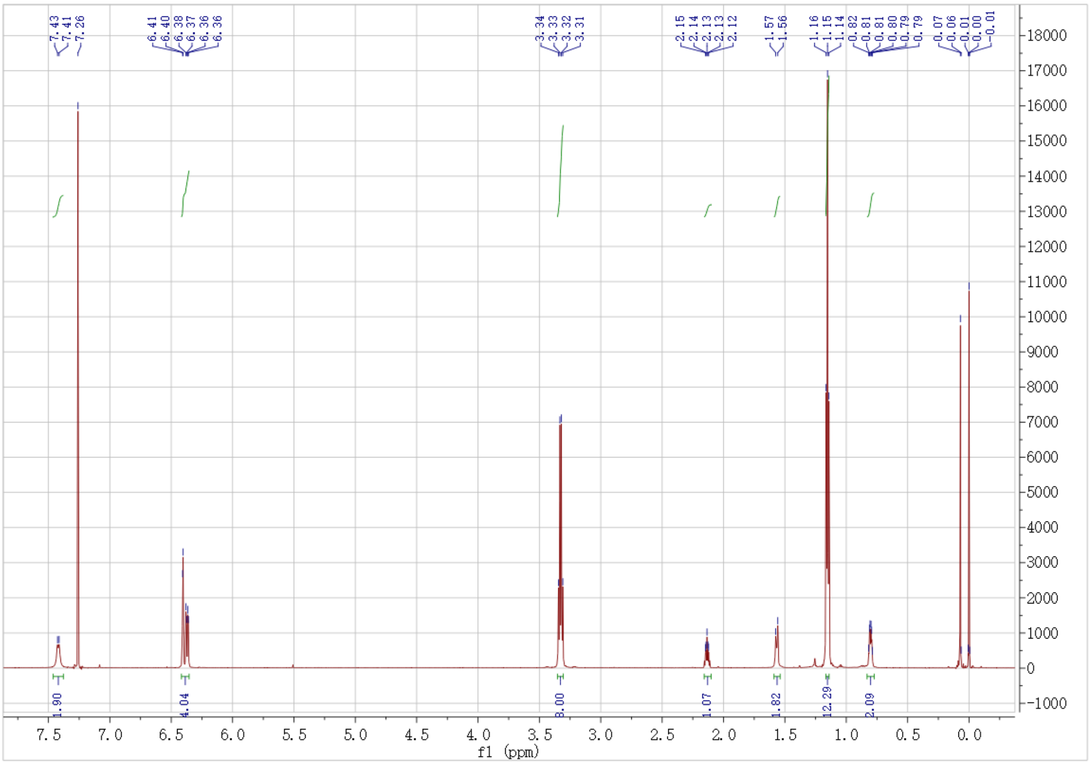

Figure S9. ^{1}H NMR of **J4**. ^{1}H NMR (600 MHz, $CDCl_3$) δ 7.42 (d, J = 7.9 Hz, 2H), 6.42 – 6.35 (m, 4H), 3.33 (q, J = 7.1 Hz, 8H), 2.16 – 2.10 (m, 1H), 1.57 (d, J = 10.0 Hz, 2H), 1.15 (t, J = 7.1 Hz, 12H), 0.80 (td, J = 6.6, 3.5 Hz, 2H).

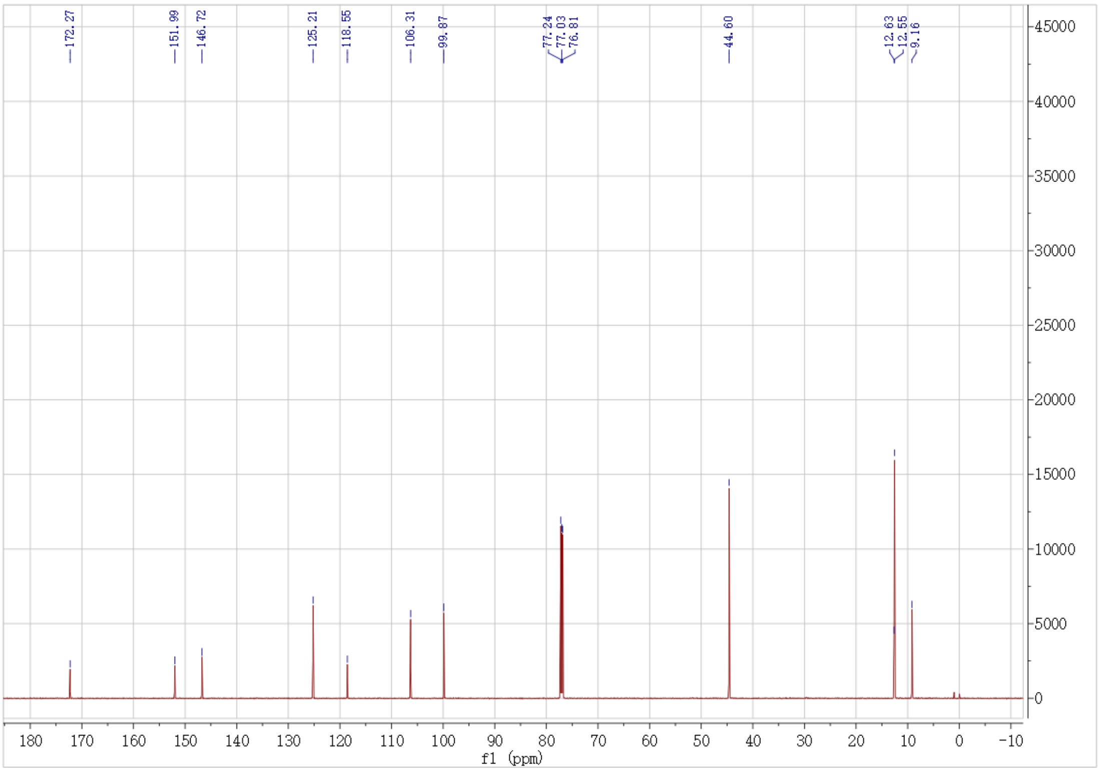

Figure S10. ^{13}C NMR spectrum of **J4** in $CDCl_3$. ^{13}C NMR (151 MHz, $CDCl_3$) δ 172.27 (s), 151.99 (s), 146.72 (s), 125.21 (s), 118.55 (s), 106.31 (s), 99.87 (s), 44.60 (s), 12.63 (s), 12.55 (s), 9.16 (s).

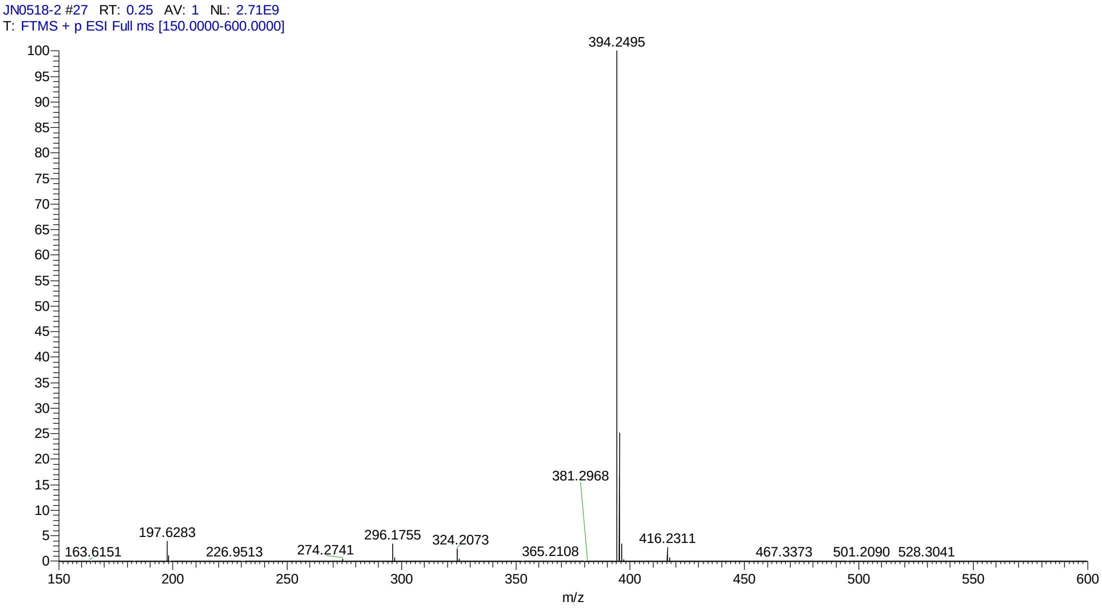

Figure S11. HR-MS of **J4**. Calc. for $C_{24}H_{32}N_3O_2^+$ [M+H]$^+$ 394.2495, found 394.2495.

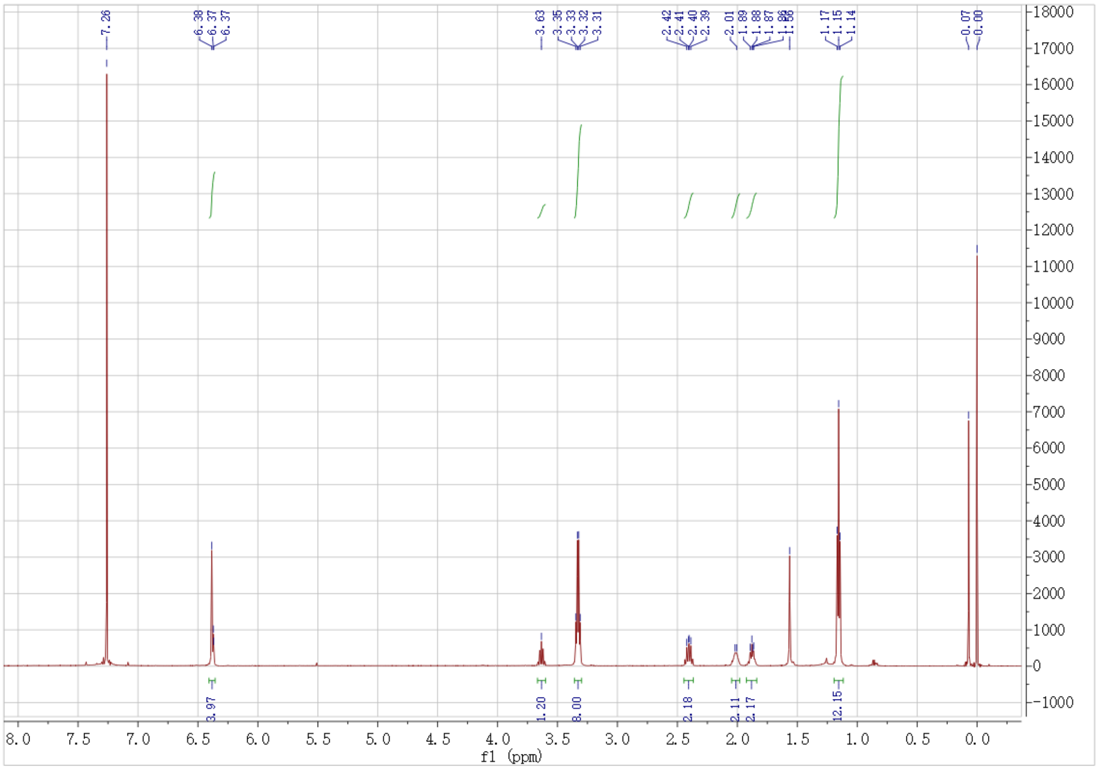

Figure S12 ^{1}H NMR spectrum of **J5** in $CDCl_3$. ^{1}H NMR (600 MHz, $CDCl_3$) δ 6.41 – 6.36 (m, 4H), 3.63 (s, 1H), 3.33 (q, J = 7.0 Hz, 8H), 2.40 (dd, J = 11.6, 8.9 Hz, 2H), 2.01 (d, J = 7.8 Hz, 2H), 1.88 (dd, J = 11.1, 6.7 Hz, 2H), 1.15 (t, J = 7.0 Hz, 12H).

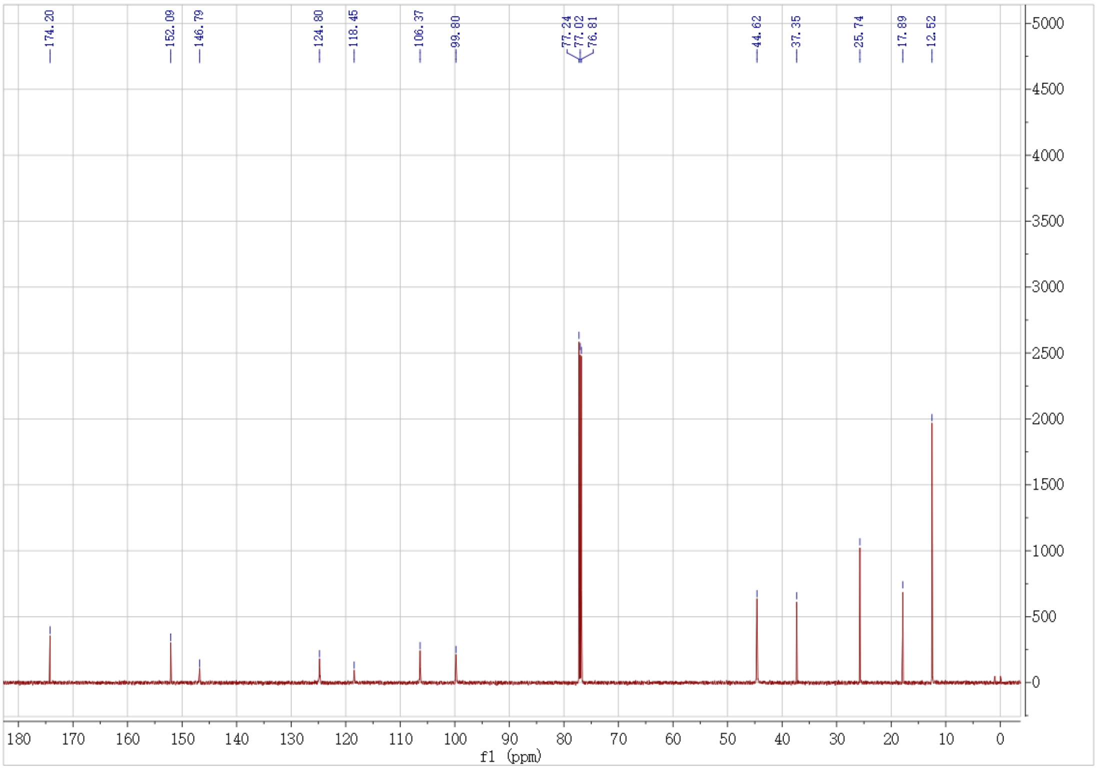

Figure S13 ^{13}C NMR spectrum of **J5** in $CDCl_3$. ^{13}C NMR (151 MHz, $CDCl_3$) δ 174.20 (s), 152.09 (s), 146.79 (s), 124.80 (s), 118.45 (s), 106.37 (s), 99.80 (s), 44.62 (s), 37.35 (s), 25.74 (s), 17.89 (s), 12.52

(s).

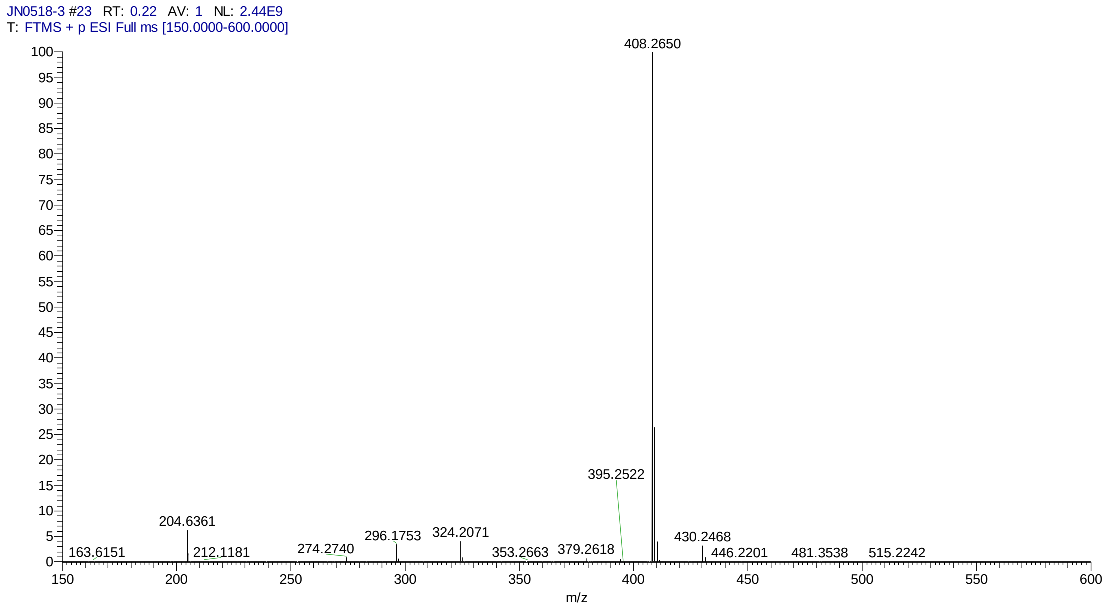

Figure S14 HR-MS of **J5**. Calc. for $C_{25}H_{34}N_3O_2^+$ $[M+H]^+$ 408.2651, found 408.2650.

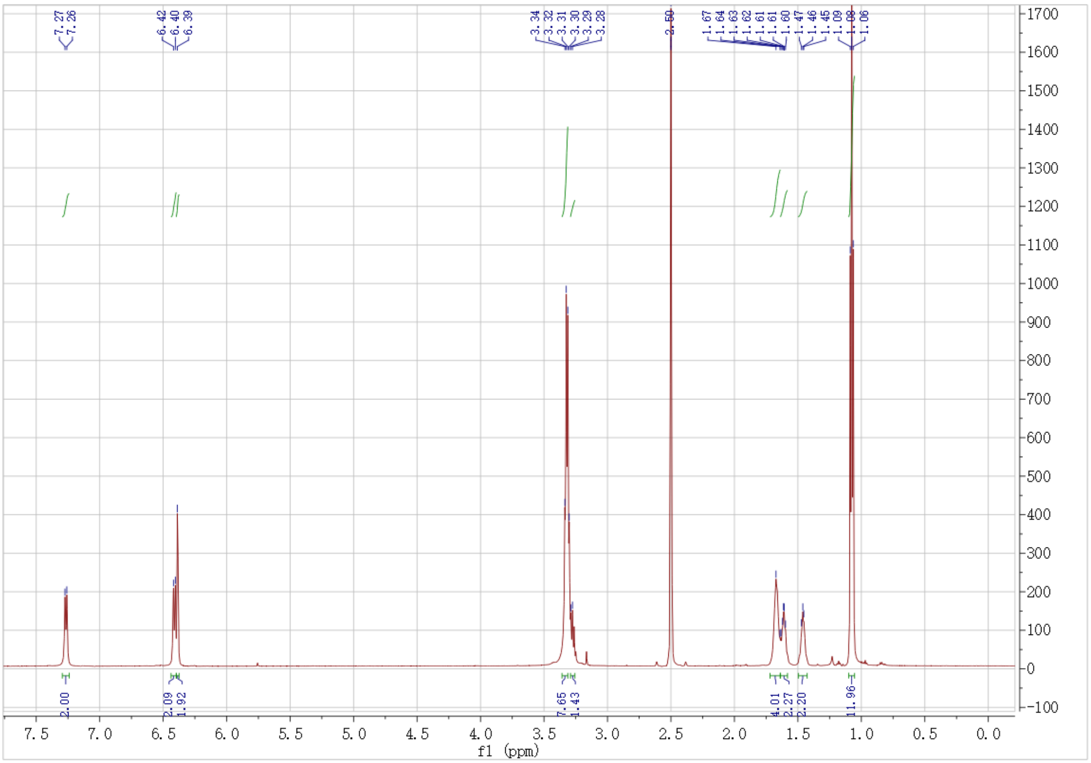

Figure S15 ^{1}H NMR spectrum of **J6** in DMSO-d_6. ^{1}H NMR (600 MHz, DMSO) δ 7.27 (d, J = 8.7 Hz, 2H), 6.41 (d, J = 8.9 Hz, 2H), 6.39 (s, 2H), 3.36 – 3.31 (m, 8H), 3.28 (d, J = 8.0 Hz, 1H), 1.66 (d, J = 18.6 Hz, 4H), 1.64 – 1.58 (m, 2H), 1.50 – 1.43 (m, 2H), 1.08 (t, J = 7.0 Hz, 12H).

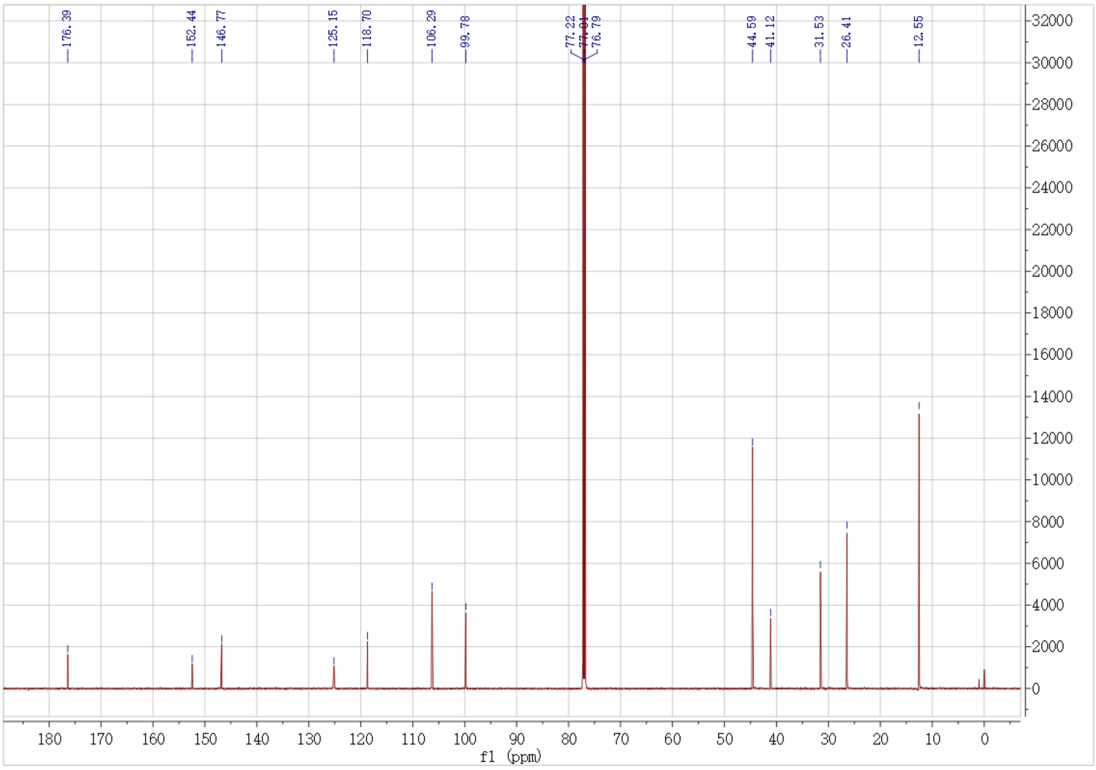

Figure S16 ^{13}C NMR spectrum of **J6** in $CDCl_3$. ^{13}C NMR (151 MHz, $CDCl_3$) δ 176.39 (s), 152.44 (s), 146.77 (s), 125.15 (s), 118.70 (s), 106.29 (s), 99.78 (s), 44.59 (s), 41.12 (s), 31.53 (s), 26.41 (s), 12.55 (s).

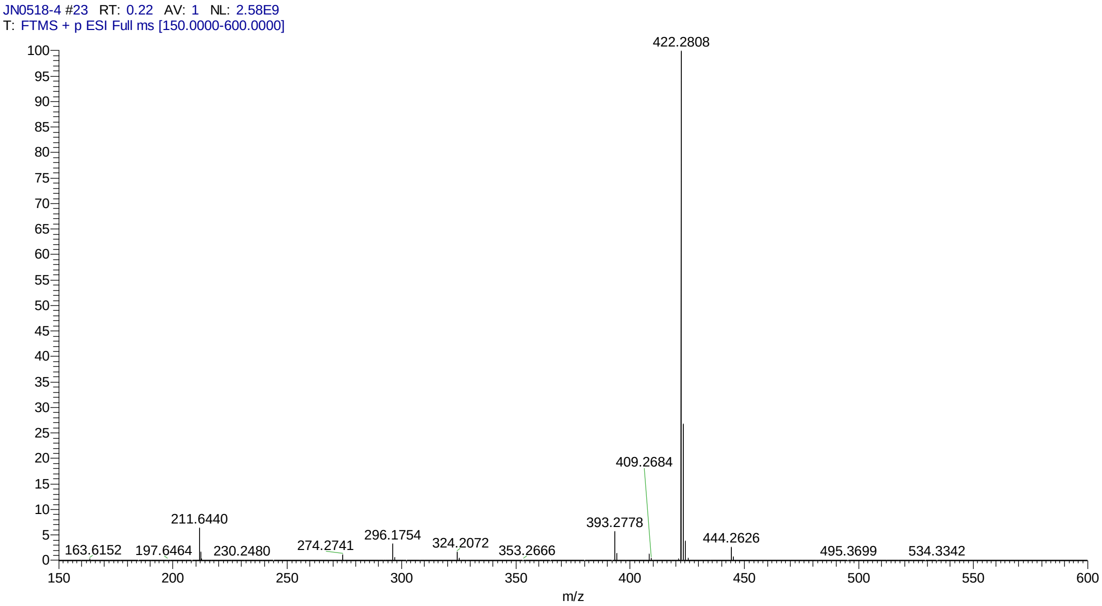

Figure S17 HR-MS of **J6**. Calc. for $C_{26}H_{36}N_3O_2^+$ $[M+H]^+$ 422.2808, found 422.2808.

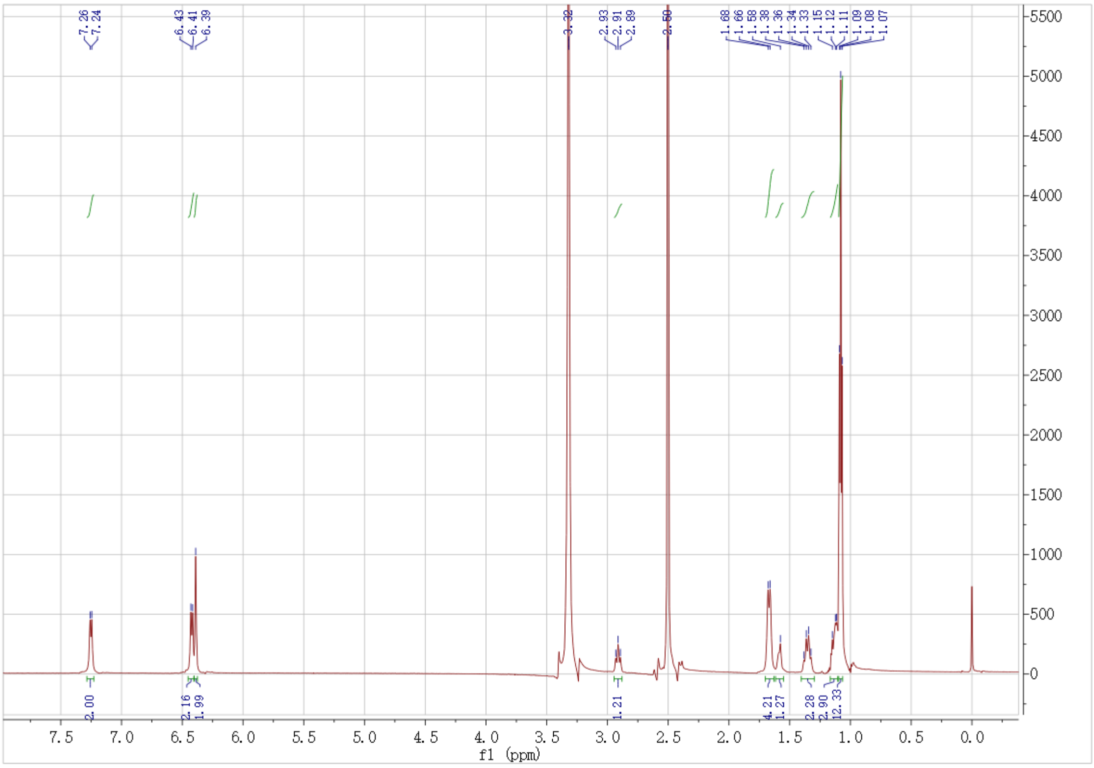

Figure S18 ^{1}H NMR spectrum of **J7** in DMSO-d_6. ^{1}H NMR (600 MHz, DMSO) δ 7.25 (d, *J* = 8.4 Hz, 2H), 6.42 (d, *J* = 8.7 Hz, 2H), 6.39 (s, 2H), 2.91 (t, *J* = 11.3 Hz, 1H), 1.67 (d, *J* = 9.9 Hz, 4H), 1.58 (s, 1H), 1.41 – 1.30 (m, 2H), 1.14 (dd, *J* = 21.4, 6.5 Hz, 3H), 1.08 (t, *J* = 6.8 Hz, 12H).

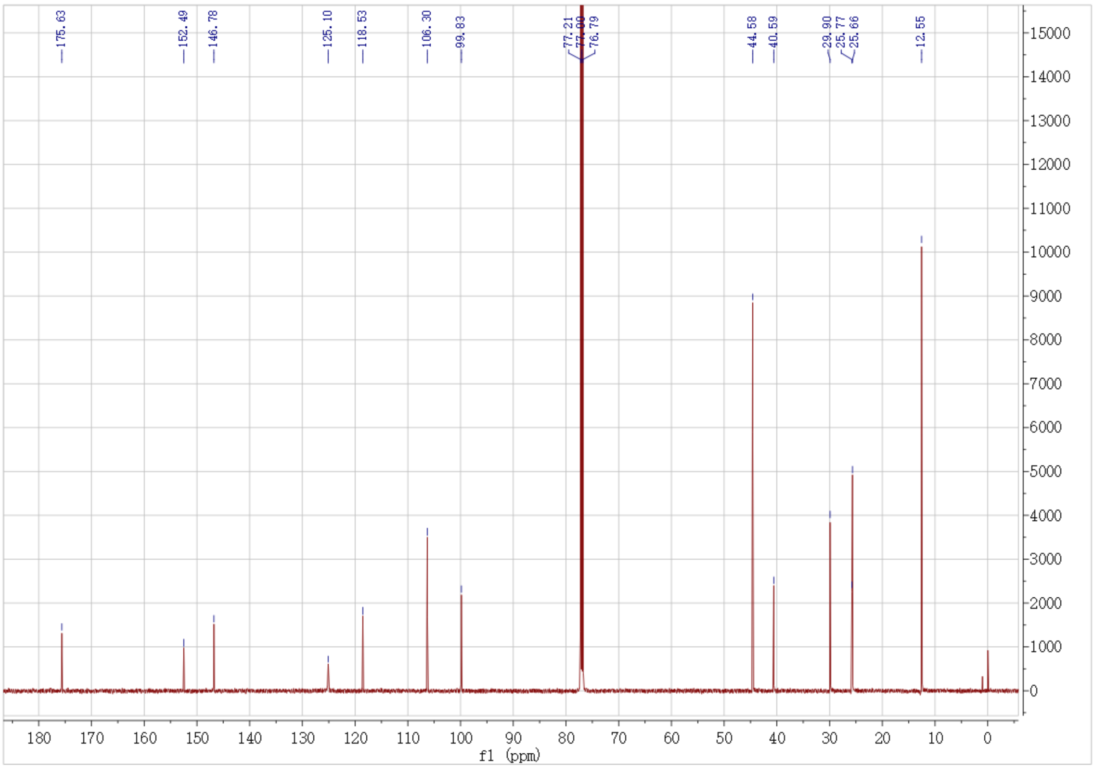

Figure S19 ^{13}C NMR spectrum of **J7** in $CDCl_3$. ^{13}C NMR (151 MHz, $CDCl_3$) δ 175.63, 152.49, 146.78, 125.10, 118.53, 106.30, 99.83, 44.58, 40.59, 29.90, 25.77, 25.66, 12.55.

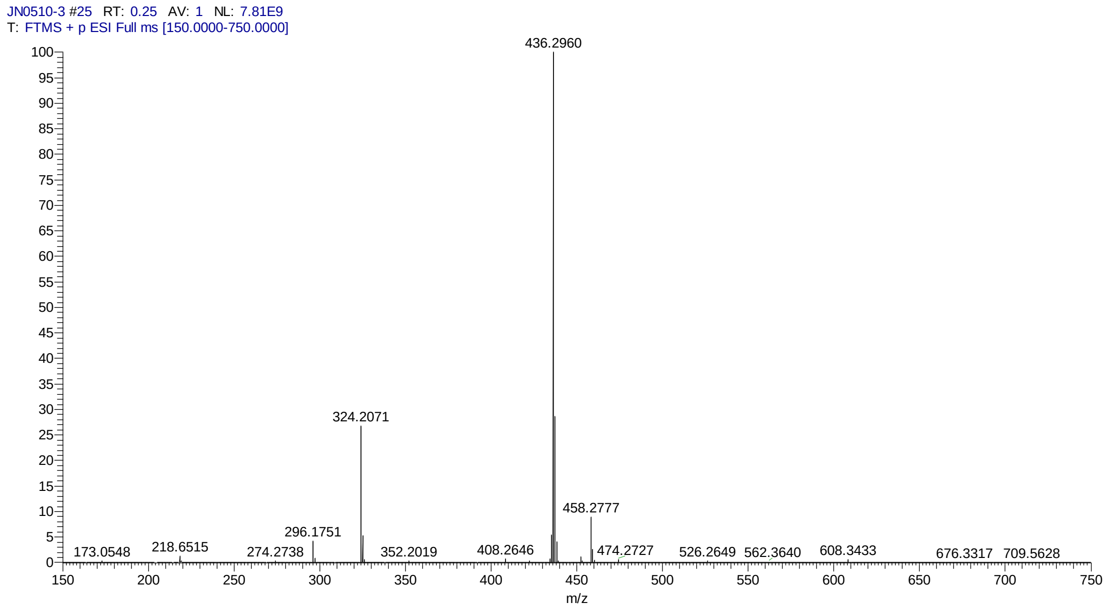

Figure S20 HR-MS of **J7**. Calc. for $C_{27}H_{38}N_3O_2^+$ $[M+H]^+$ 436.2964, found 436.2960.

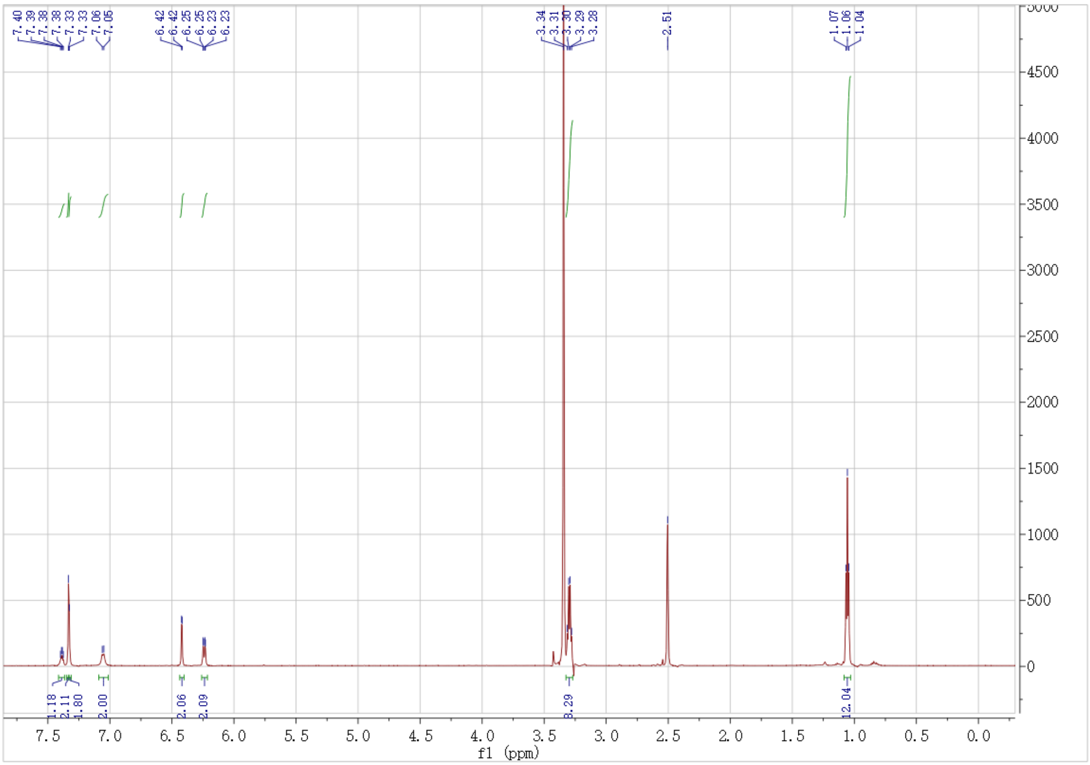

Figure S21 ^{1}H NMR spectrum of **J11** in DMSO-d_6. ^{1}H NMR (600 MHz, DMSO) δ 7.39 (dd, J = 8.8, 4.7 Hz, 1H), 7.33 (s, 2H), 7.33 (s, 2H), 7.06 (d, J = 7.9 Hz, 2H), 6.42 (d, J = 2.5 Hz, 2H), 6.24 (dd, J = 8.9, 2.2 Hz, 2H), 3.30 (q, J = 6.8 Hz, 8H), 1.06 (t, J = 7.0 Hz, 12H).

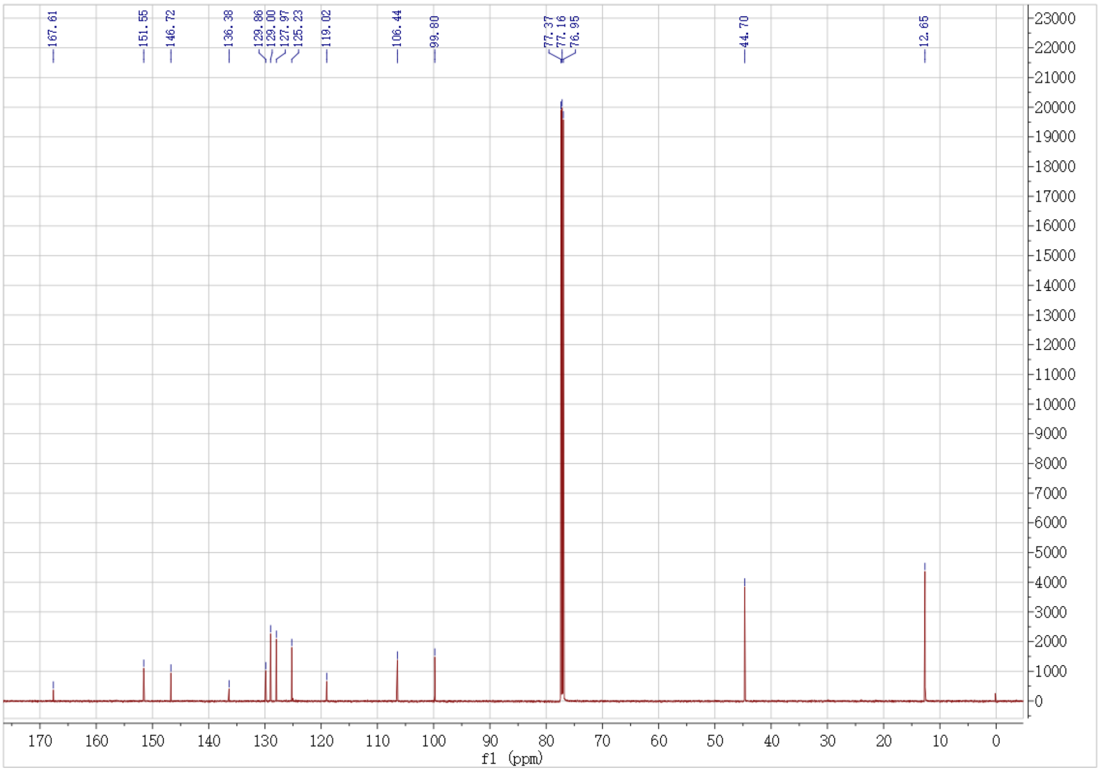

Figure S22 ^{13}C NMR spectrum of **J11** in $CDCl_3$. ^{13}C NMR (151 MHz, $CDCl_3$) δ 167.61, 151.55, 146.72, 136.38, 129.86, 129.00, 127.97, 125.23, 119.02, 106.44, 99.80, 77.37, 77.16, 76.95, 44.70, 12.65.

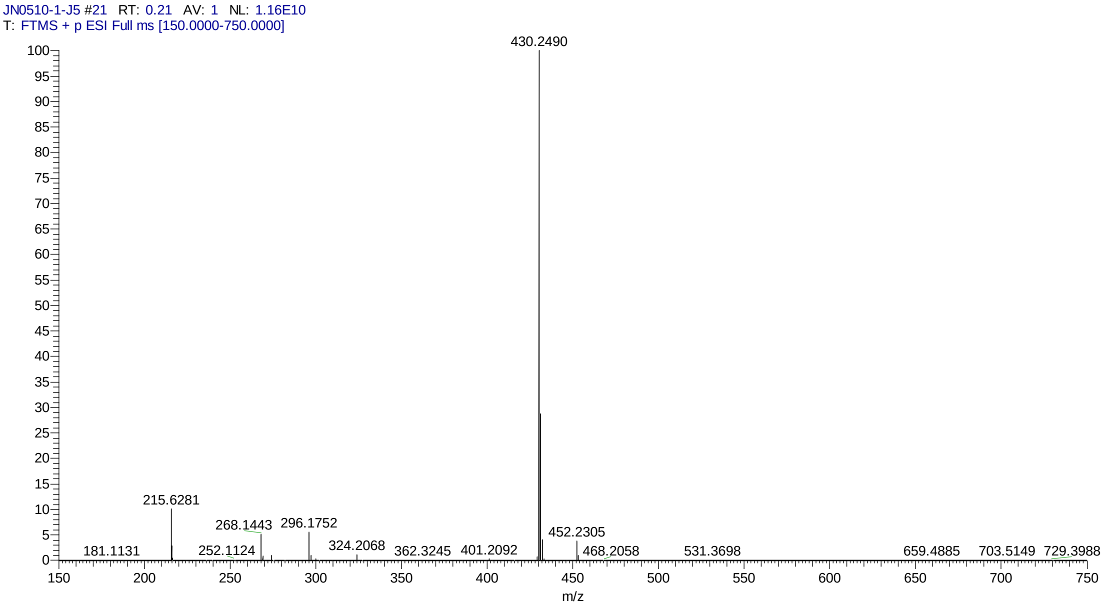

Figure S23 HR-MS of **J11**. Calc. for $C_{27}H_{32}N_3O_2^+$ $[M+H]^+$ 430.2495, found 430.2490.

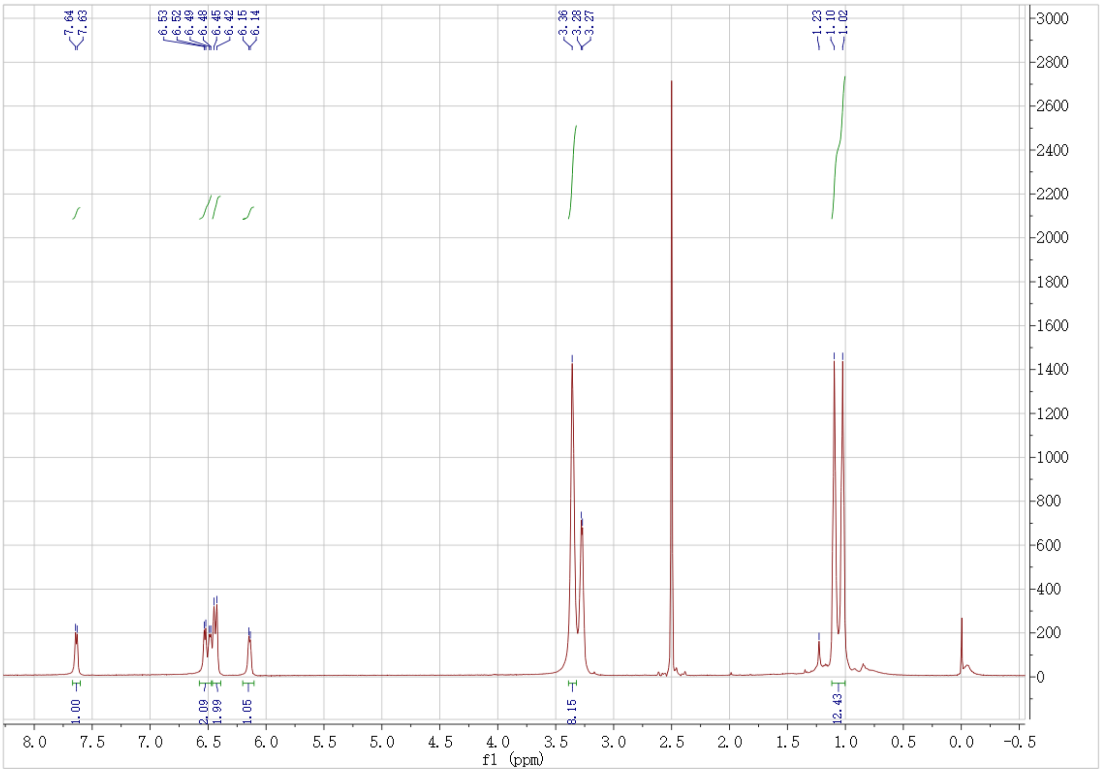

Figure S24 ^{1}H NMR spectrum of **J12** in DMSO-d_6. ^{1}H NMR (600 MHz, DMSO) δ 7.64 (d, J = 8.1 Hz, 1H), 6.50 (dd, J = 26.6, 7.7 Hz, 2H), 6.44 (d, J = 14.9 Hz, 2H), 6.14 (d, J = 7.4 Hz, 1H), 3.36 (s, 8H), 1.06 (d, J = 43.9 Hz, 12H).

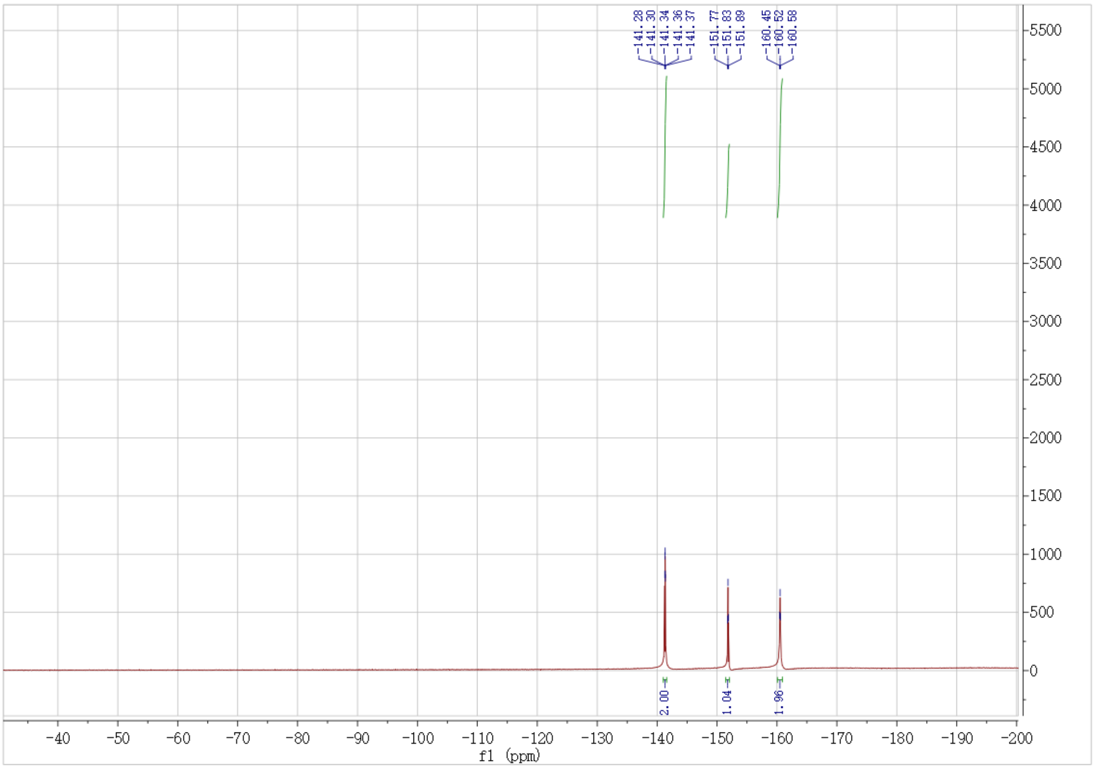

Figure S25 ^{19}F NMR spectrum of **J12** in DMSO-d_6. ^{19}F NMR (376 MHz, DMSO-d_6) δ -141.03 – -141.59 (m, 2F), -151.83 (t, J = 23.3 Hz, 1F), -160.08 – -160.91 (m, 2F).

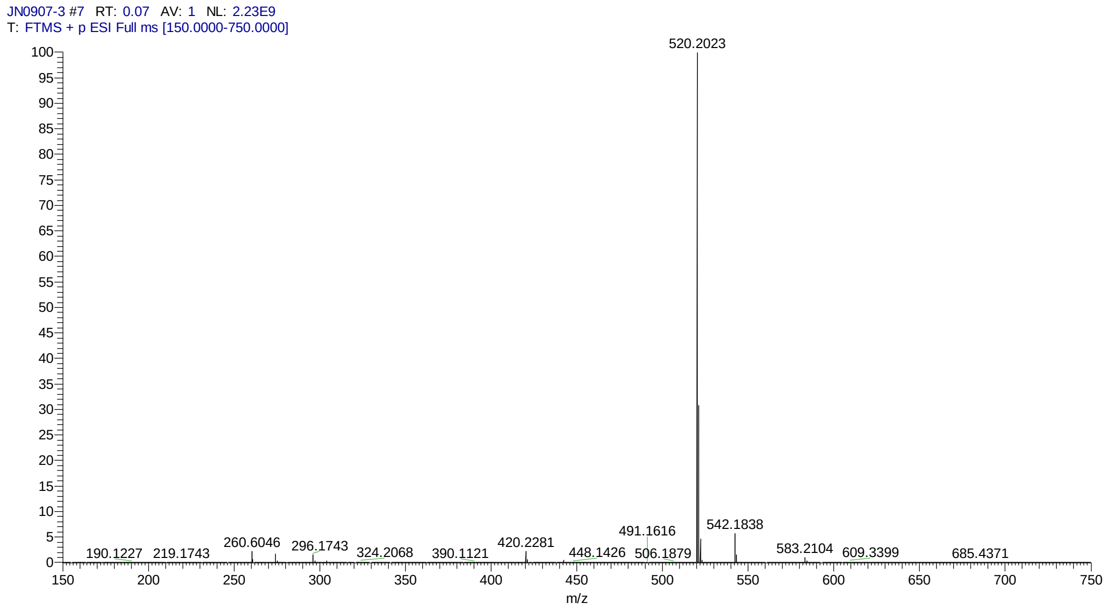

Figure S26 HR-MS of **J12**. Calc. for $C_{27}H_{27}FN_3O_2^+$ $[M+H]^+$ 520.2023, found 520.2023.

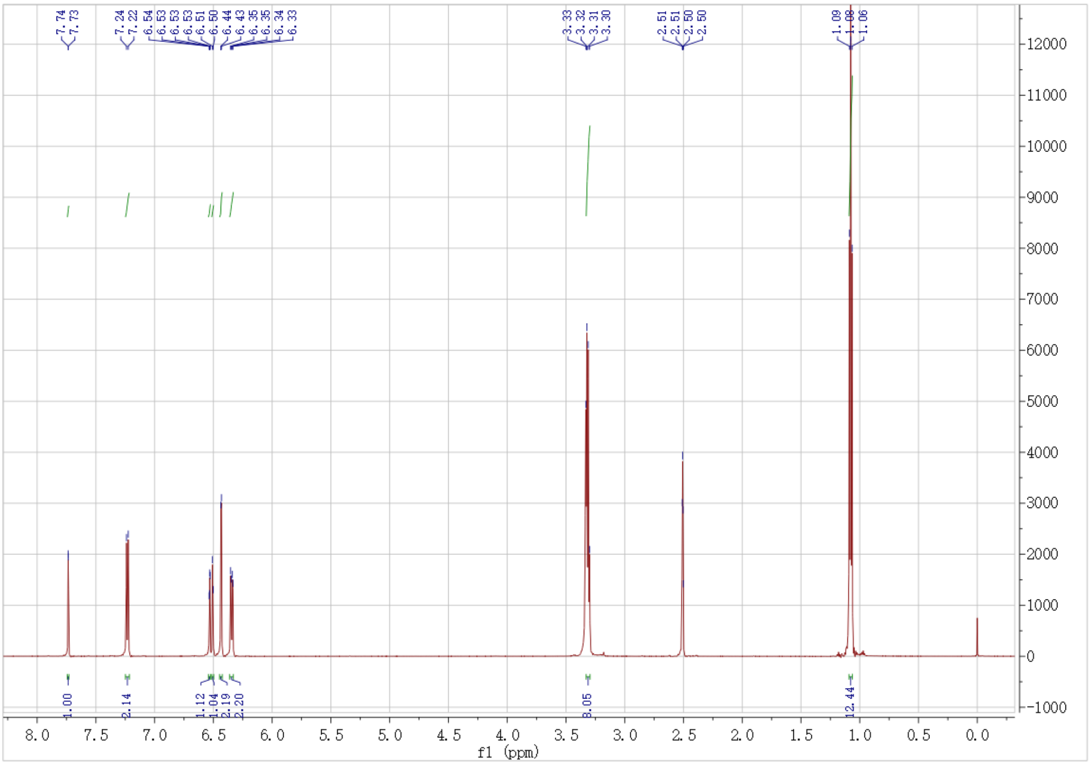

Figure S27 ^{1}H NMR spectrum of $\mathbf{J_{Fast}}$ in DMSO-d_6. ^{1}H NMR (600 MHz, DMSO) δ 7.74 (d, J = 0.9 Hz, 1H), 7.23 (d, J = 9.0 Hz, 2H), 6.53 (dd, J = 3.5, 1.7 Hz, 1H), 6.51 (d, J = 3.4 Hz, 1H), 6.43 (d, J = 2.7 Hz, 2H), 6.34 (dd, J = 9.0, 2.7 Hz, 2H), 3.33 – 3.29 (m, 8H), 1.08 (t, J = 7.0 Hz, 12H).

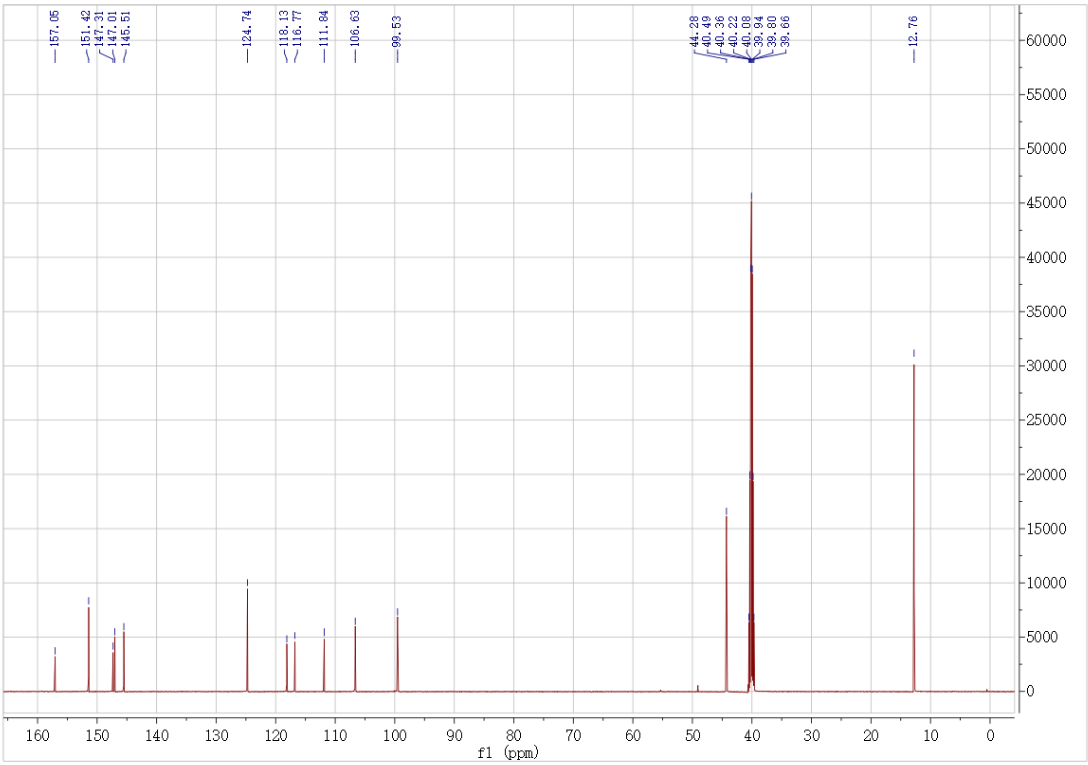

Figure S28 ^{13}C NMR spectrum of $\mathbf{J_{Fast}}$ in DMSO-d_6. ^{13}C NMR (151 MHz, DMSO) δ 157.05 (s), 151.42 (s), 147.31 (s), 147.01 (s), 145.51 (s), 124.74 (s), 118.13 (s), 116.77 (s), 111.84 (s), 106.63 (s), 99.53 (s), 44.28 (s), 40.42 (d, J = 20.9 Hz), 40.22 (s), 40.22 (s), 40.08 (s), 39.94 (s), 39.80 (s), 39.66 (s), 12.76 (s).

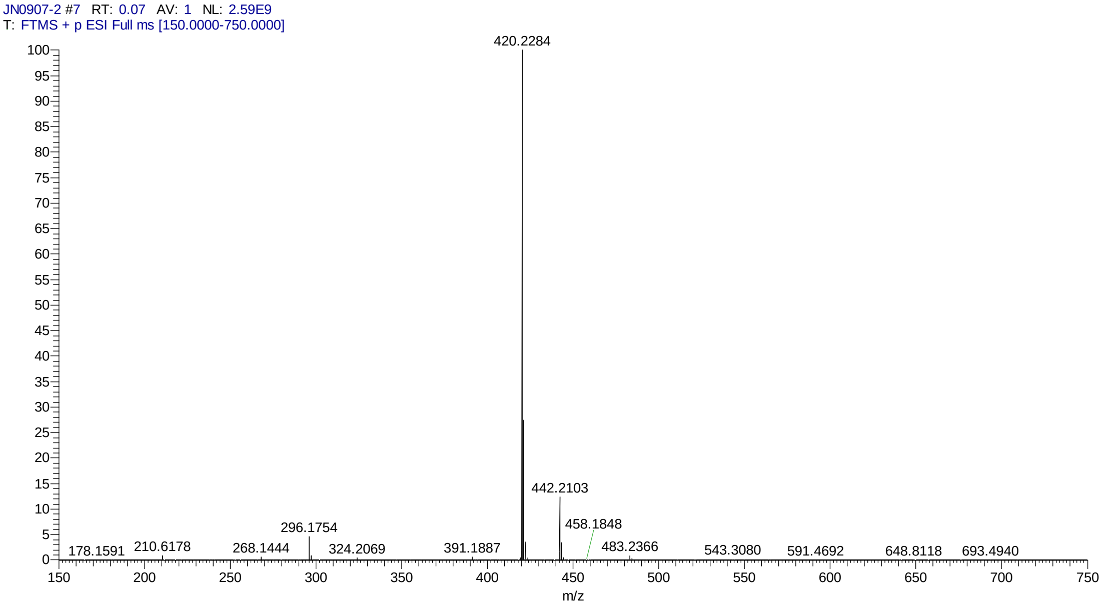

Figure S29HR-MS of $\mathbf{J_{Fast}}$. Calc. for $C_{25}H_{30}N_3O_3^+$ $[M+H]^+$ 420.2287, found 420.2284.

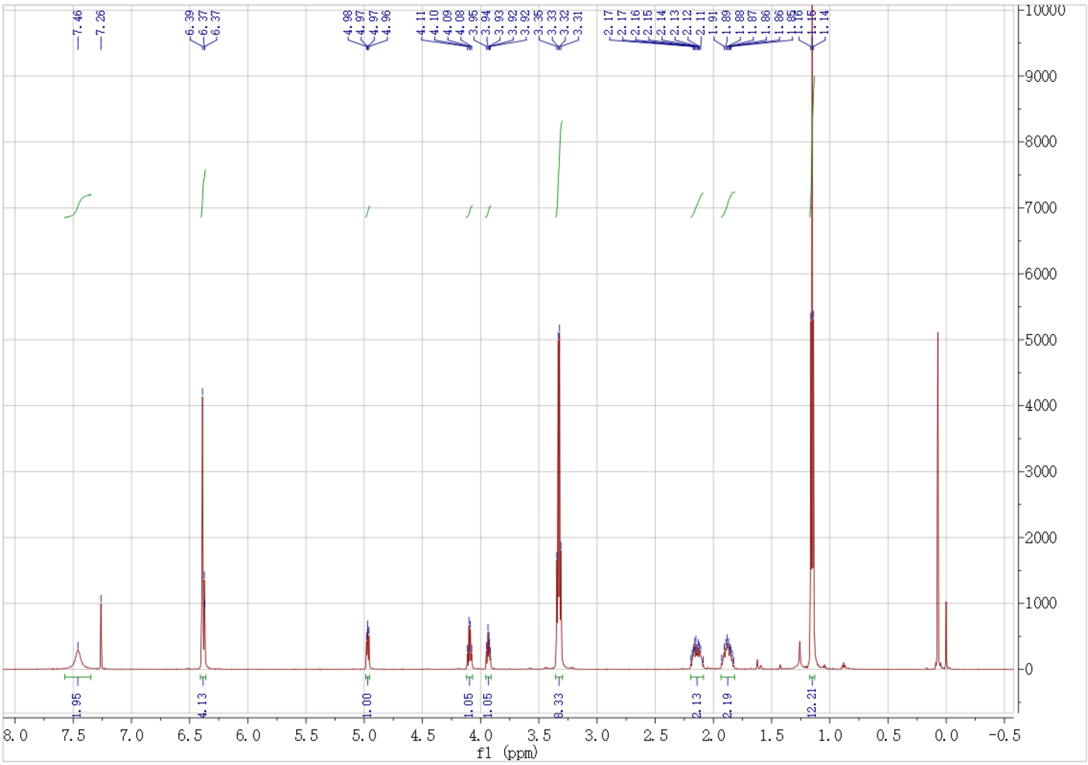

Figure S30 ^{1}H NMR spectrum of **J8** in $CDCl_3$. ^{1}H NMR (600 MHz, $CDCl_3$) δ 7.46 (s, 2H), 6.40 – 6.36 (m, 4H), 4.97 (dd, J = 7.3, 5.3 Hz, 1H), 4.09 (d, J = 7.3 Hz, 1H), 3.93 (dd, J = 10.2, 5.0 Hz, 1H), 3.33 (q, J = 7.0 Hz, 8H), 2.20 – 2.08 (m, 2H), 1.87 (ddd, J = 14.4, 13.1, 7.5 Hz, 2H), 1.15 (t, J = 7.0 Hz, 12H).

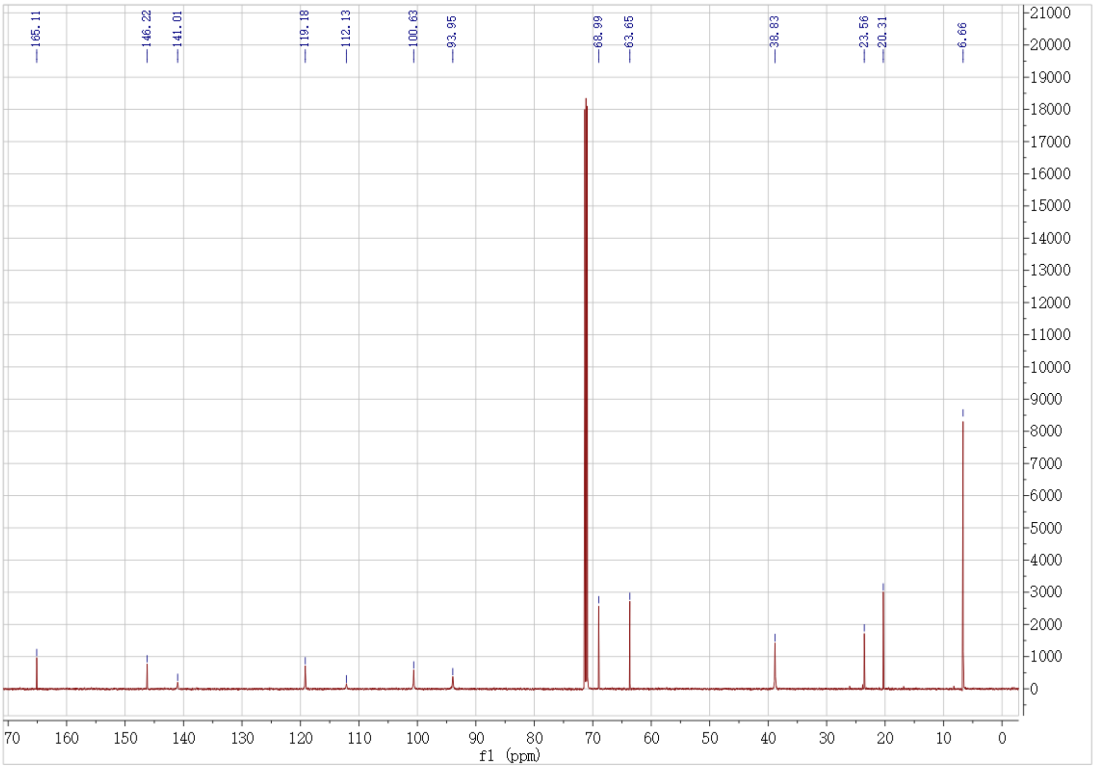

Figure S31 ^{13}C NMR spectrum of **J8** in $CDCl_3$. ^{13}C NMR (151 MHz, $CDCl_3$) δ 165.11 (s), 146.22 (s),

141.01 (s), 119.18 (s), 112.13 (s), 100.63 (s), 93.95 (s), 68.99 (s), 63.65 (s), 38.83 (s), 23.56 (s), 20.31 (s), 6.66 (s).

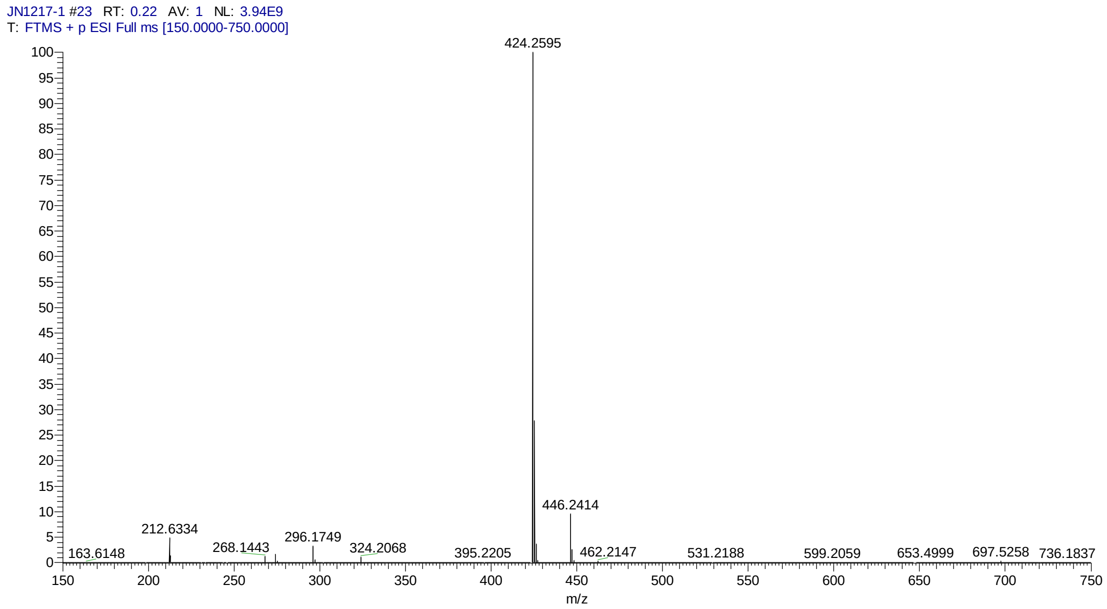

Figure S32 HR-MS of **J8**. Calc. for $C_{25}H_{34}N_3O_3^+$ $[M+H]^+$ 424.2600, found 424.2595.

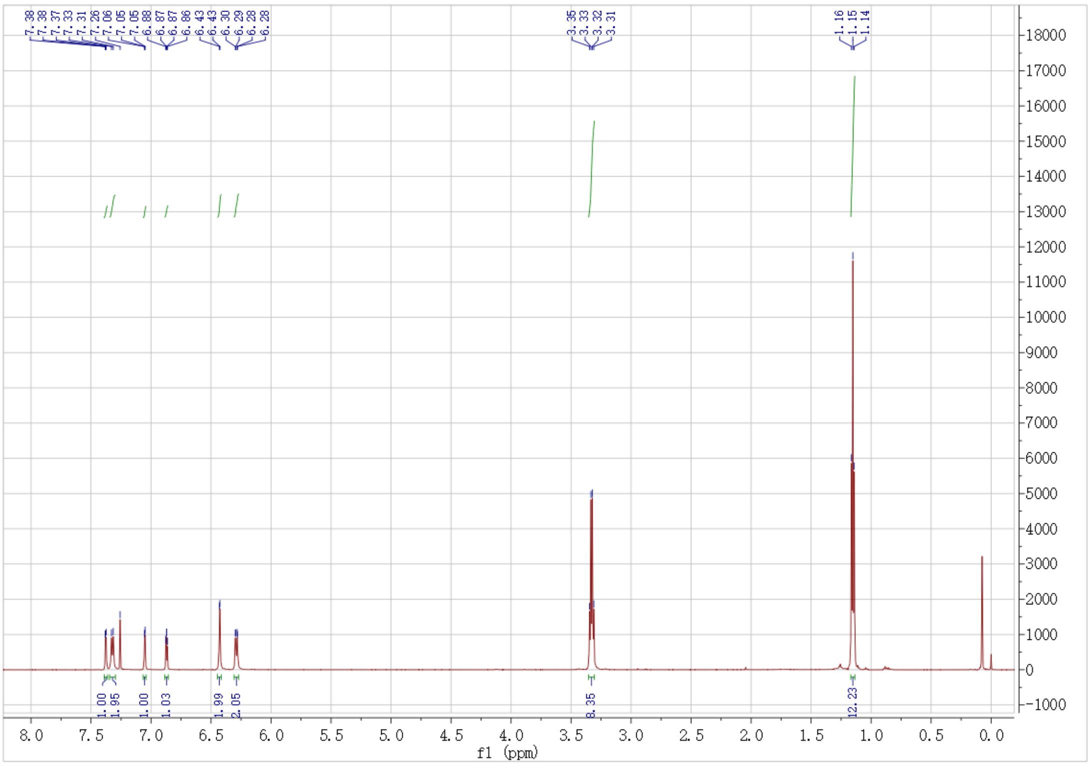

Figure S33 ^{1}H NMR spectrum of **J9** in $CDCl_3$. ^{1}H NMR (600 MHz, $CDCl_3$) δ 7.39 – 7.36 (m, 1H), 7.32 (d, J = 8.9 Hz, 2H), 7.07 – 7.04 (m, 1H), 6.87 (dd, J = 4.7, 4.0 Hz, 1H), 6.43 (d, J = 2.2 Hz, 2H), 6.29 (dd, J = 8.9, 2.3 Hz, 2H), 3.33 (q, J = 7.0 Hz, 8H), 1.15 (t, J = 7.1 Hz, 12H).

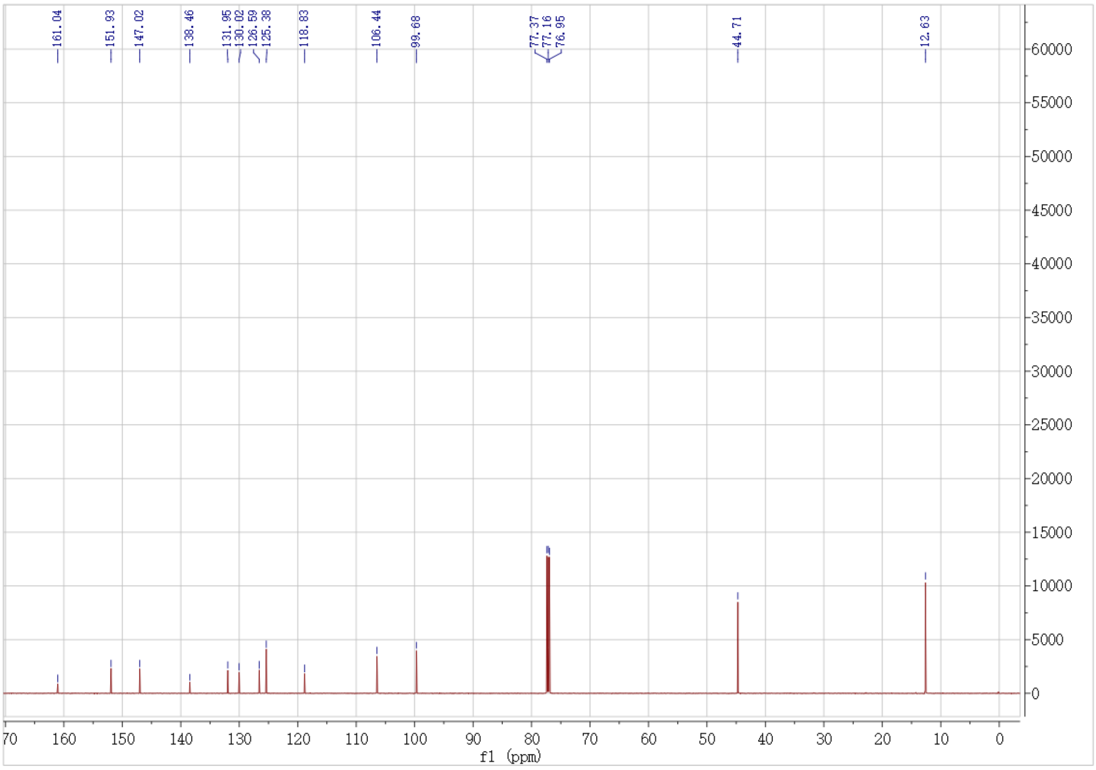

Figure S34 ^{13}C NMR spectrum of **J9** in $CDCl_3$. ^{13}C NMR (151 MHz, $CDCl_3$) δ 161.04, 151.93, 147.02, 138.46, 131.95, 130.02, 126.59, 125.38, 118.83, 106.44, 99.68, 44.71, 12.63.

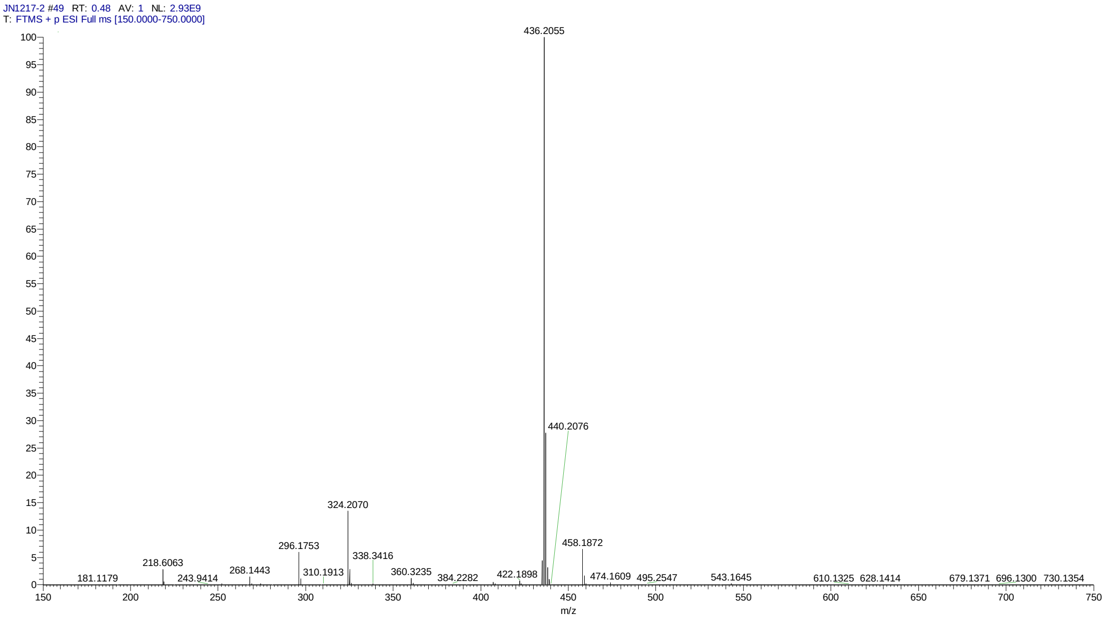

Figure S35 HR-MS of **J9**. Calc. for $C_{25}H_{30}N_3O_2S^+$ $[M+H]^+$ 436.2059, found 436.2055.

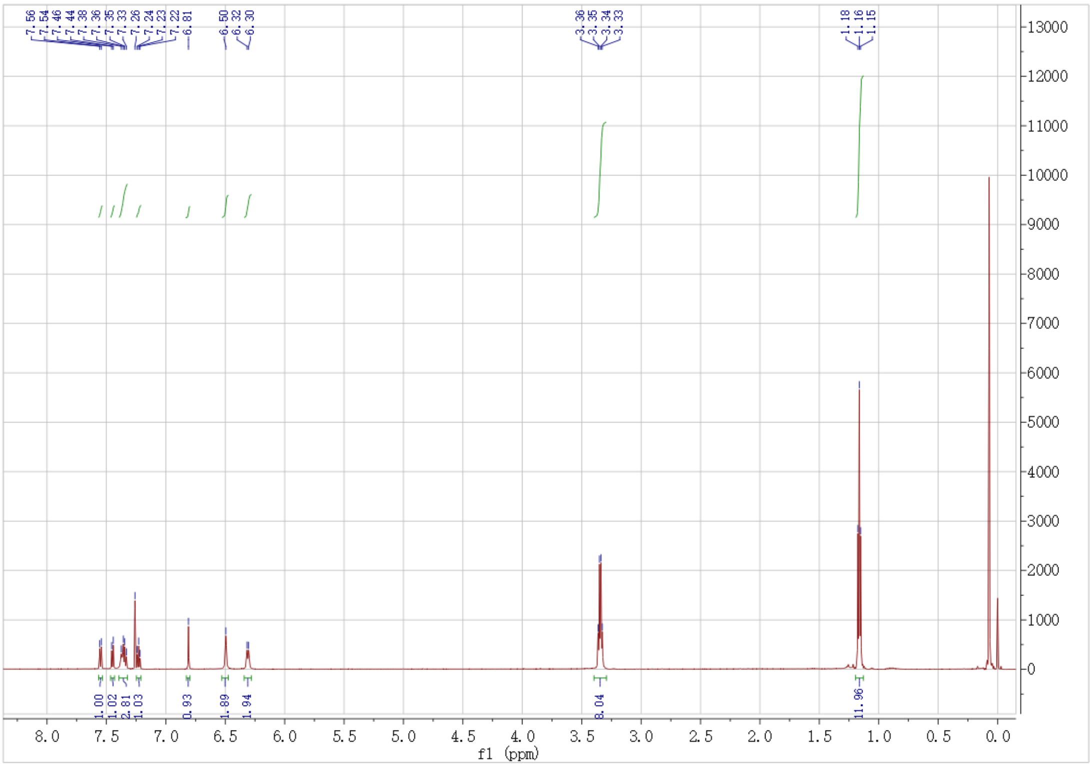

Figure S36 ^{1}H NMR spectrum of **J10** in $CDCl_3$. ^{1}H NMR (600 MHz, $CDCl_3$) δ 7.55 (d, J = 7.8 Hz, 1H), 7.45 (d, J = 8.3 Hz, 1H), 7.35 (dd, J = 16.7, 9.2 Hz, 3H), 7.23 (t, J = 7.5 Hz, 1H), 6.81 (s, 1H), 6.50 (s, 2H), 6.31 (d, J = 8.1 Hz, 2H), 3.34 (q, J = 7.0 Hz, 8H), 1.16 (t, J = 7.0 Hz, 12H).

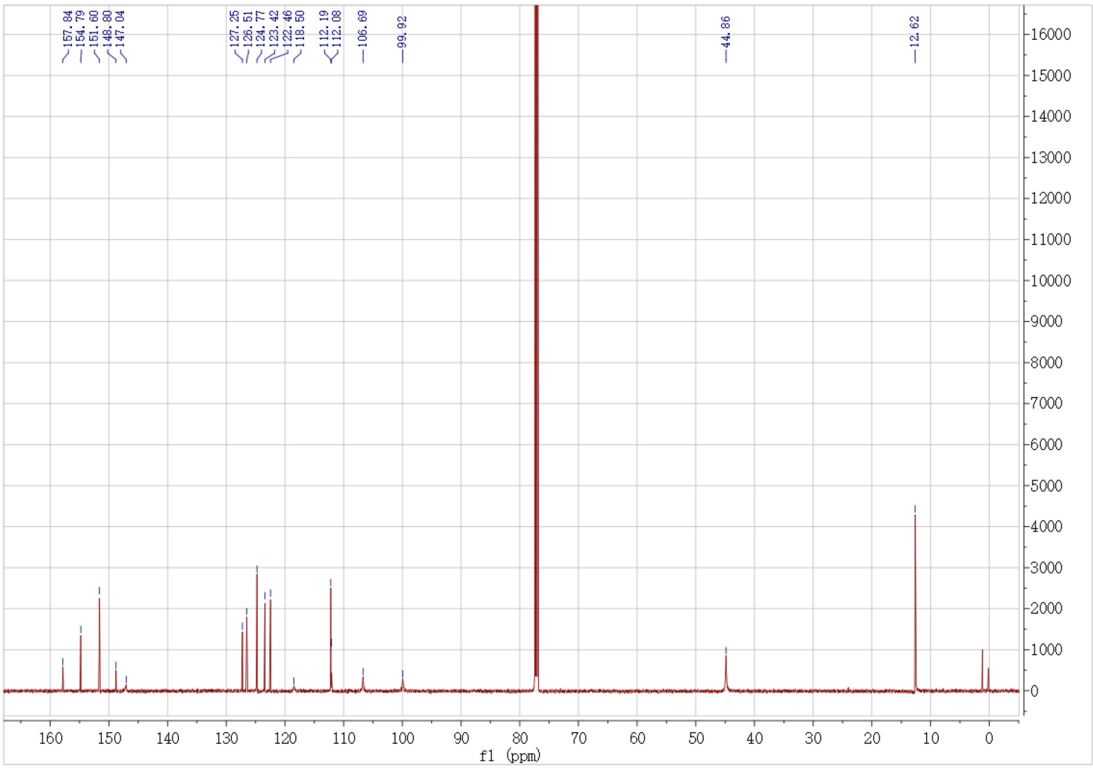

Figure S37 ^{13}C NMR spectrum of **J10** in $CDCl_3$. ^{13}C NMR (151 MHz, $CDCl_3$) δ 157.84, 154.79, 151.60, 148.80, 147.04, 127.25, 126.51, 124.77, 123.42, 122.46, 118.50, 112.19, 112.08, 106.69, 99.92,

44.86, 12.62.

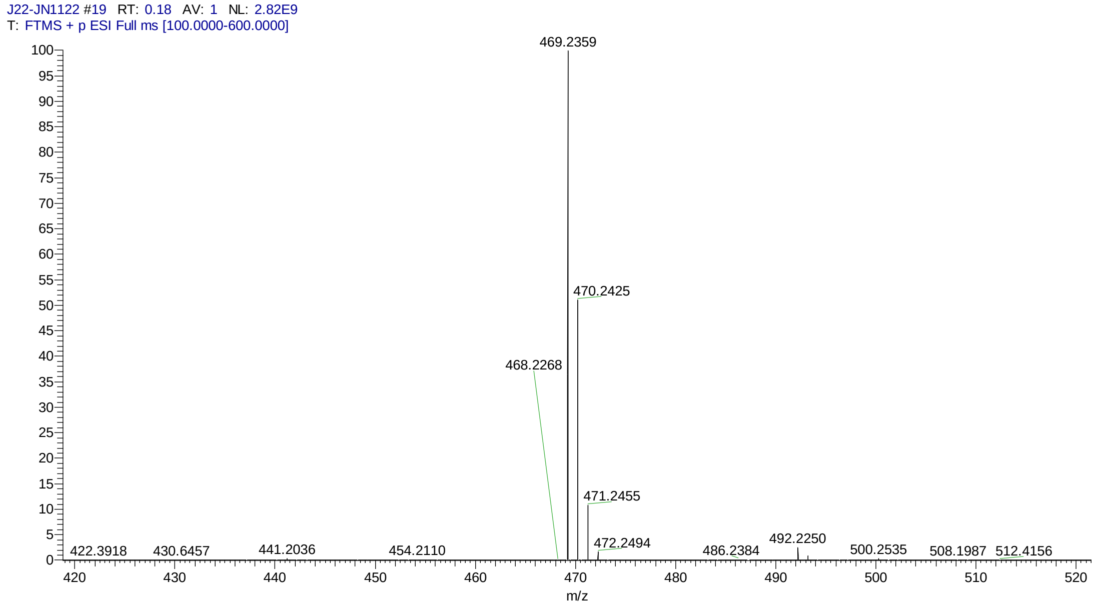

Figure S38 HR-MS of **J10**. Calc. for $C_{29}H_{32}N_3O_3^+$ $[M+H]^+$ 470.2444, found 470.2359.

4. Additional Fluorescence Tests

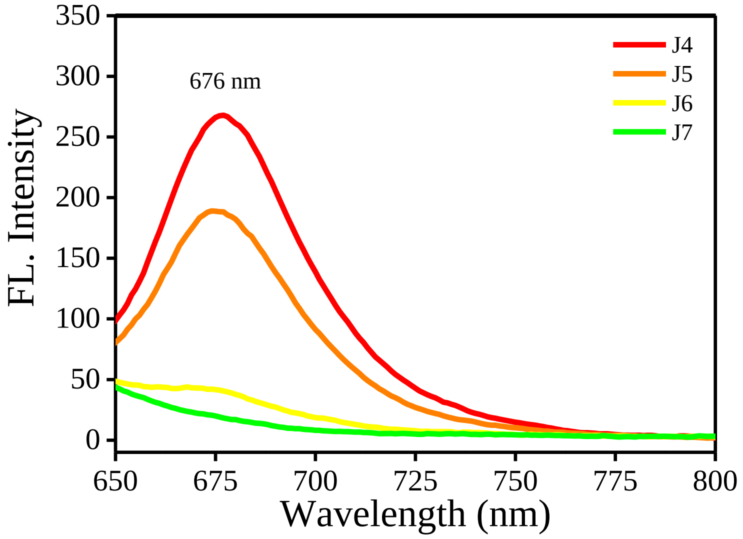

Figure S39. Fluorescence spectral of **J4-J7** (5 μM) upon addition of CE (10 U/mL) in PBS (pH 7.4) after 120 min, λ_{ex} = 630 nm, Slit: 5 nm/5 nm.

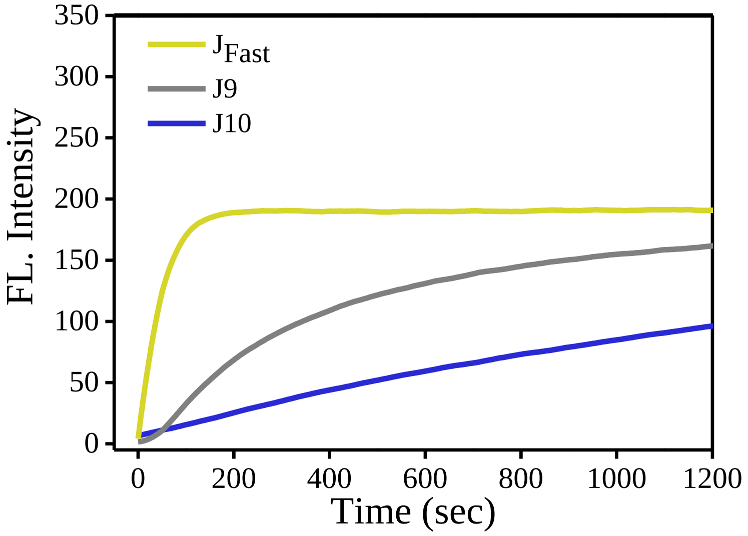

Figure S40. Time-dependent change of fluorescence emission intensity (λ_{ex} = 630 nm, λ_{ex} = 676 nm) of probes (5 μM) upon addition with CE (10 U/mL).

5. Additional Results derived from Molecular Docking Simulations

Table S2. The docking coefficient, DistSer221, and DistHis468 derived from the molecular modeling simulations of these probes with the crystal complex of human CES1.

**Probes**	**Substituent Structure**	**Energy (kcal/mol)**	**DistSer221 (Å)**	**DistHis468 (Å)**
**J1**	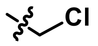	50.47	7.2	9.0
**J2**	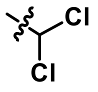	50.80	6.2	7.2
**J3**	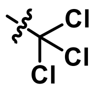	55.38	6.3	7.5
**J4**	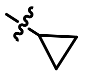	53.78	6.5	8.3
**J5**	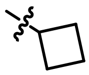	51.79	6.6	7.9

**J6**	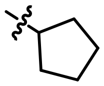	56.61	6.7	8.0
**J7**	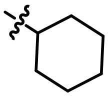	60.04	4.0	5.1
**J$_{Fast}$**	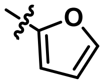	56.61	4.0	5.0
**J8**	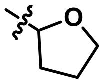	50.36	7.1	8.4
**J9**	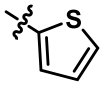	55.63	5.8	5.9
**J10**	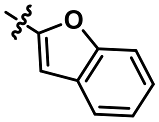	55.63	6.4	7.6
**J11**	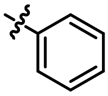	56.01	4.0	4.9
**J12**	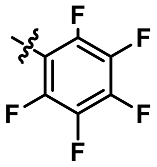	58.39	4.1	5.0

*Note: DistSer221and DistHis468: The distance between the amide group of probes and Ser^{221} and His^{468}, respectively. Energy: This value is obtained from -Cdocker Interaction calculated.

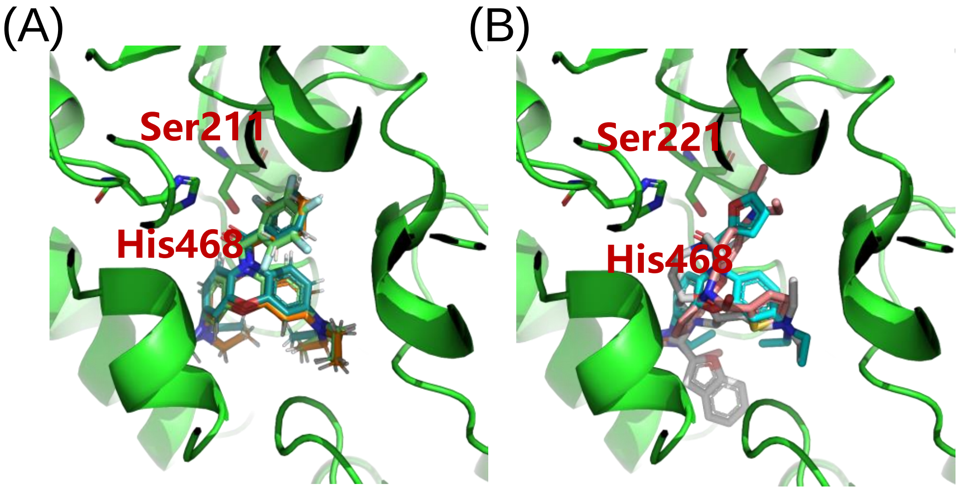

Figure S41. Docked structures highlighting the interaction of probes in the active site of CE. In (A), **J7**, **J11** and **J12** structures are shown in orange, black green and green, respectively. In (B), $\mathbf{J_{Fast}}$, **J9** and **J10** structures are shown in cyan, salmon, and gray, respectively, respectively.

6. Additional Fluorescence Tests in Cell Lysate.

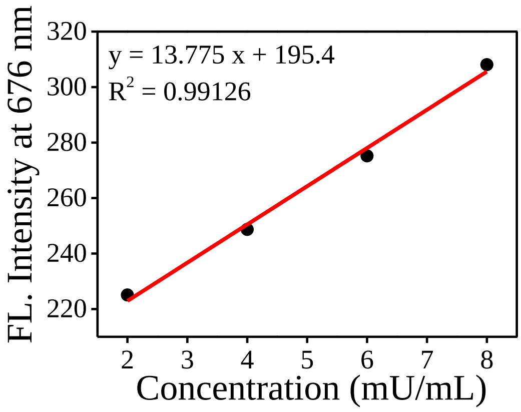

Figure S42. Plot of the fluorescence intensity of the $\mathbf{J_{Fast}}$ as a function of the CE concentration in cell lysate, upon excitation at 630 nm.

7. Additional Fluorescence Imaging in Living Cells

We detected the CE activity in co-culture model (HepG2 and HL7702 cells), as shown in Figure S43. Because the morphological differences between the two types of cells were not obvious, HL7702 cells were specifically labelled with 5 μM **NP1** dyes for 30 min (**NP1**, as a specific probe for H_2O_2, was reported in our previous work; *Anal. Chem.*, 2014, 86, 9970-9976), before co-incubation with HepG2 cells. Then the adherent cells were incubated with 5 μM $\mathbf{J_{Fast}}$ for 30 min. In the blue channel (445 ± 20 nm, λ_{ex} = 405 nm), HL7702 cells can be verified (shown in yellow box, Figure S43), due to **NP1**

staining. While a bright red fluorescence signal was monitored in HepG2 cells in white box. These data indicated that the probe may serve as a tool to distinguish normal cells (HL7702 cells) and cancer cells (HepG2 cells).

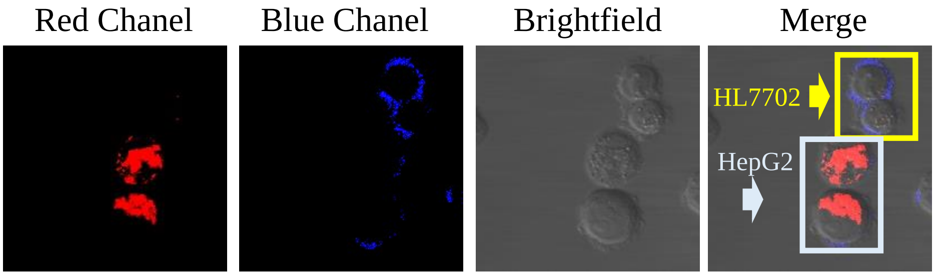

Figure S43. CLSM images of co-culture HepG2 and HL7702 cells incubated with $\mathbf{J_{Fast}}$. Before co-incubation with HepG2 cells, HL7702 cells were specifically labelled with 5 µM **NP1** dyes for 30 min (**NP1**, as a specific probe for H_2O_2, was reported in our previous work; *Anal. Chem.*, 2014, 86, 9970-9976). Red channel: 680±30 nm, λ_{ex} = 633 nm, for $\mathbf{J_{Fast}}$; Blue channel: 445 ± 20 nm, λ_{ex} = 405 nm, for **NP1**; scale bar = 10 µm.

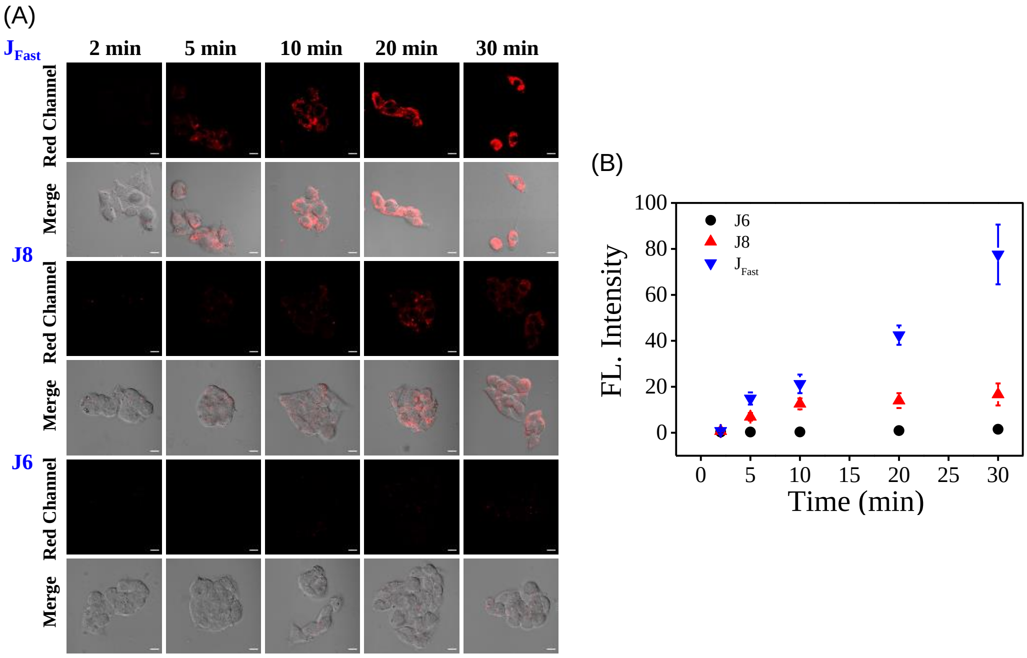

Figure S44. (A) Time-dependent CLSM of CE activity in HepG2 cells incubation with $\mathbf{J_{Fast}}$, **J8** and **J6** (10 µM). (B). Crresponding average fluorescence intensities of HepG2 cells in red channels in (A).

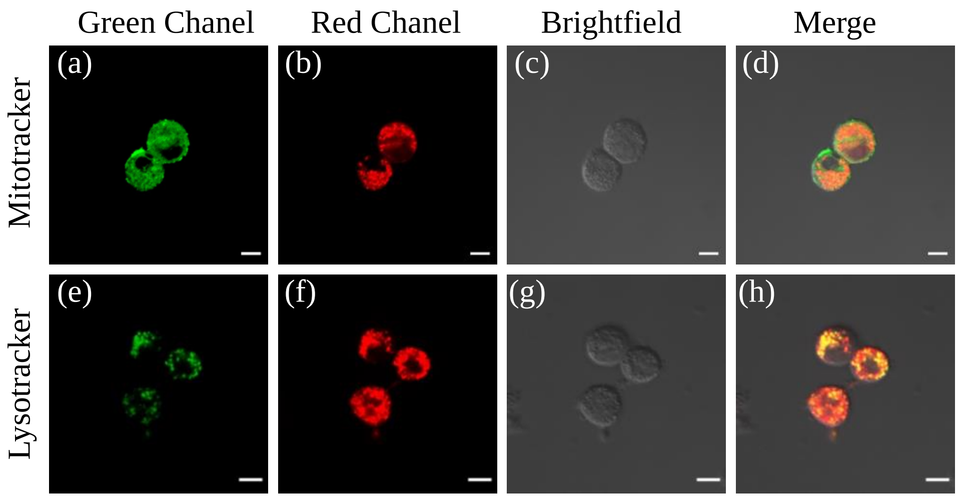

Figure S45. CLSM images of HepG2 cells co-labeled with (a-d) $\mathbf{J}_{\mathbf{Fast}}$ (5 μM)/Mitotracker (500 nM), and (e-h) $\mathbf{J}_{\mathbf{Fast}}$ (5 μM)/Lysotracker (1 μM) at 37 °C. Red channel: 680±30 nm, λ_{ex} = 633 nm, for $\mathbf{J}_{\mathbf{Fast}}$; Green channel: 530 ± 20 nm, λ_{ex} = 488 nm, for Mitotracker or Lysotracker; Scale bar = 10 μm.

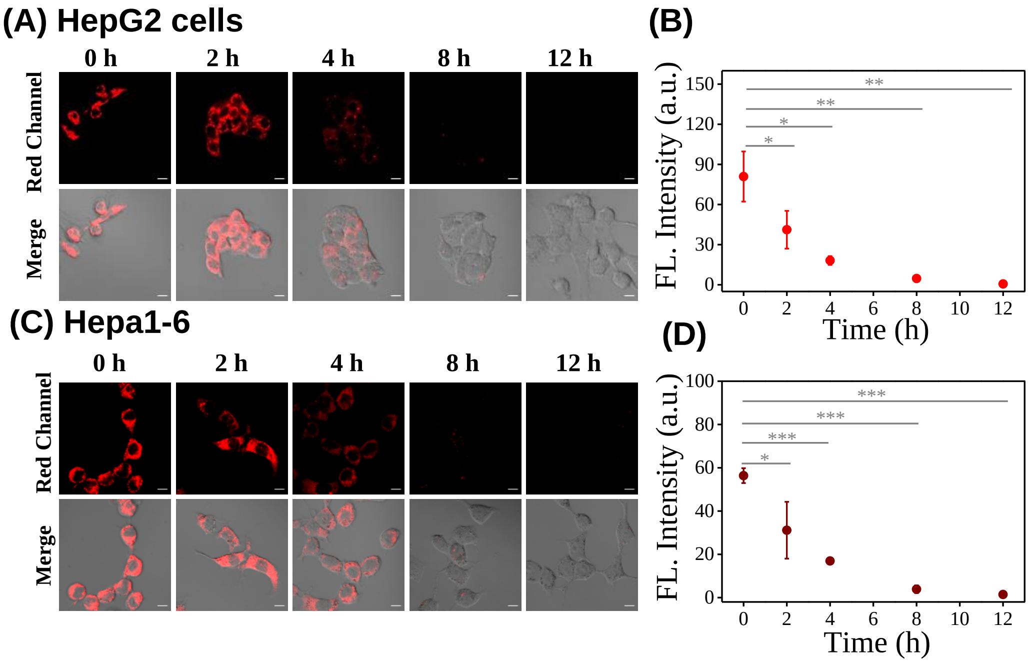

Figure S46. CLSM images of $\mathbf{J}_{\mathbf{Fast}}$-loaded (A) HepG2 cells or (C) Hepa1-6 cells (10 μM, 30 min) pre-treatment with sorafenib (10 μM) for different times (0, 2, 4, 8 and 12 h). Red channel: 680±30 nm, λ_{ex} = 633 nm; scale bar = 10 μm. (B) and (D) Corresponding average fluorescence intensities of HepG2 cells or Hepa1-6 cells in red channels in (A) or (D), respectively. *p < 0.05, **p < 0.01, ***p < 0.001, error bars are ± S.D.

8. Cell Viability

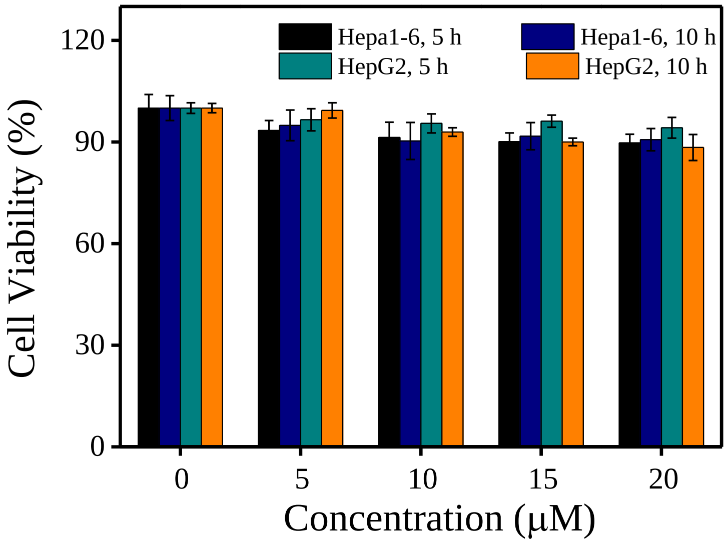

Figure S47. Cell viability values (%) estimated by CCK-8 assay in HepG2 and Hepa1-6 cells, which were cultured in the presence of 0-20 μM $\mathbf{J_{Fast}}$ for 5 and 10 h.

9. Additional Fluorescence Imaging in *in vivo*

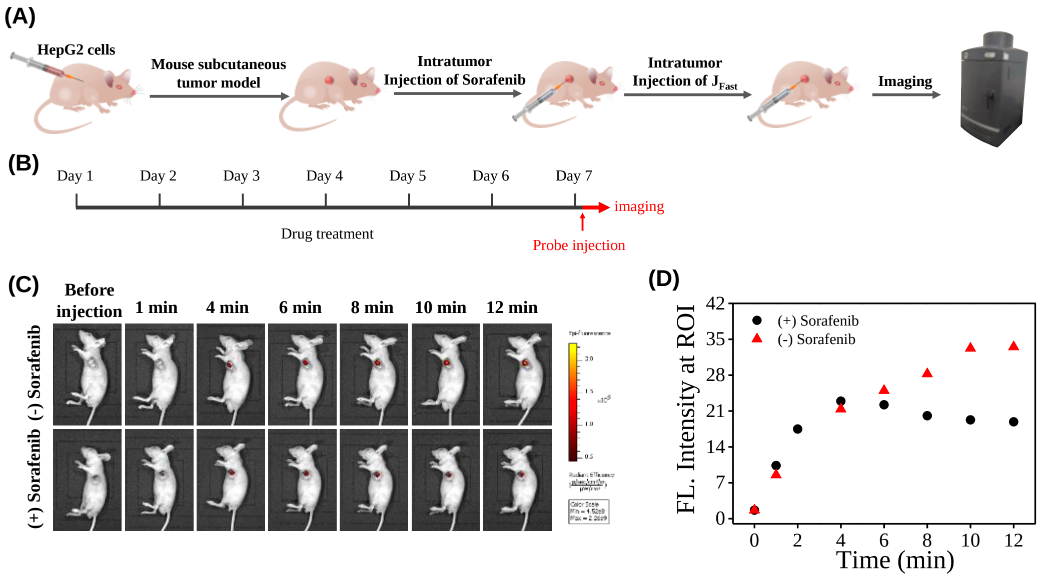

Figure S48. (A) Schematic illustration of $\mathbf{J_{Fast}}$ imaging CE activity in subcutaneous HepG2-xenografted tumor-bearing mouse model treated with anticancer drug (sorafenib). (B) Timeline for the development of mouse model treated with sorafenib and bimodal imaging. (C) Imaging CE activity in tumor-bearing mice through intratumor injection of $\mathbf{J_{Fast}}$ (200 μM, 20 μL) without or with

treatment by sorafenib (200 μM, continuous for one week). (D) Corresponding average fluorescence intensities of tumor region in red channels in (C). Red channel: 695-770 nm, λ_{ex} = 640 nm.

10. References

S1 Wu X., Shen Y., Pmn inhibits colorectal cancer cells through inducing mitotic arrest and p53-dependent apoptosis via the inhibition of tubulin polymerization. *Biochem. Biophys. Res. Commun.,* **2018**, 499, 927-933.

S2 Qi Y. L., Wang H. R., Chen L. L., et al., Multifunctional fluorescent probe for simultaneously detecting microviscosity, micropolarity, and carboxylesterases and its application in bioimaging. *Anal. Chem.,* **2022**, 94, 4594-4601.

S3 Zhang X. Y., Liu T. T., Liang J. H., et al., A highly selective near infrared fluorescent probe for carboxylesterase 2 and its biological applications. *J. Mater. Chem. B,* **2021**, 9, 2457-2461.

S4 Liu S. Y., Qu R. Y., Li R. R., et al., An activity-based fluorogenic probe enables cellular and in vivo profiling of carboxylesterase isozymes. *Anal. Chem.,* **2020**, 92, 9205-9213.

S5 Chen P., Kuang W., Zheng Z., et al., Carboxylesterase-cleavable biotinylated nanoparticle for tumor-dual targeted imaging. *Theranostics,* **2019**, 9, 7359-7369.

S6 Ding L., Tian Z., Hou J., et al., Sensing carboxylesterase 1 in living systems by a practical and isoform-specific fluorescent probe. *Chin. Chem. Lett.,* **2019**, 30, 558-562.

S7 Tian Z., Ding L., Li K., et al., Rational design of a long-wavelength fluorescent probe for highly selective sensing of carboxylesterase 1 in living systems. *Anal. Chem.,* **2019**, 91, 5638-5645.

S8 Park S. J., Kim Y. J., Kang J. S., et al., Carboxylesterase-2-selective two-photon ratiometric probe reveals decreased carboxylesterase-2 activity in breast cancer cells. *Anal. Chem.,* **2018**, 90, 9465-9471.

S9 Wang J., Chen Q., Tian N., et al., A fast responsive, highly selective and light-up fluorescent probe for the two-photon imaging of carboxylesterase in living cells. *J. Mater. Chem. B,* **2018**, 6, 1595-1599.

S10 Wang Y., Ma C., Zheng X., et al., A red emission multiple detection site probe for detecting carboxylesterase 1 based on bodipy fluorophore. *J. Photochem. Photobiol. A,* **2021**, 421.

S11 Wu X., Wang R., Qi S., et al., Rational design of a highly selective near-infrared two-photon fluorogenic probe for imaging orthotopic hepatocellular carcinoma chemotherapy. *Angew. Chem. Int. Ed. Engl.,* **2021**, 60, 15418-15425.

S12 Jiang A., Chen G., Xu J., et al., Ratiometric two-photon fluorescent probe for in situ imaging of carboxylesterase (CE)-mediated mitochondrial acidification during medication. *Chem. Commun.,* **2019**, 55, 11358-11361.

S13 Zhou H., Tang J., Zhang J., et al., A red lysosome-targeted fluorescent probe for carboxylesterase detection and bioimaging. *J. Mater. Chem. B,* **2019**, 7, 2989-2996.

S14 Wang Y., Yu F., Luo X., et al., Visualization of carboxylesterase 2 with a near-infrared two-photon fluorescent probe and potential evaluation of its anticancer drug effects in an orthotopic colon carcinoma mice model. *Chem. Commun.,* **2020**, 56, 4412-4415.

S15 Ma C., Wu J., Sun W., et al., A near infrared bodipy-based lysosome targeting probe for selectively detection of carboxylesterase 1 in living cells pretreated with pesticides. *Sens. Actuators B,* **2020**, 325.

S16 Wei P., Liu L., Wen Y., et al., Release of amino- or carboxy-containing compounds triggered by HOCl: Application for imaging and drug design. *Angew. Chem. Int. Ed.,* **2019**, 58, 4547-4551.
S17 Wei P., Yuan W., Xue F., et al., Deformylation reaction-based probe for in vivo imaging of HOCl. *Chem. Sci.,* **2018**, 9, 495-501.
S18 Wen Y., Long Z., Bai X., et al., Specific fluorescence release based on synergistic activation of enzymes and position-dependent of electrophilic groups to diagnose intrahepatic cholestasis of pregnancy. *Chem. Eng. J.*, **2022**, 440.
